# ZNF330/NOA36 interacts with HSPA1 and HSPA8 and modulates cell cycle and proliferation in response to heat shock in HEK293 cells

**DOI:** 10.1186/s13062-023-00384-8

**Published:** 2023-05-30

**Authors:** Alejandra Sanchez-Briñas, Carmen Duran-Ruiz, Antonio Astola, Marta Marina Arroyo, Fátima G. Raposo, Antonio Valle, Jorge Bolivar

**Affiliations:** 1 Department of Biomedicine, Biotechnology and Public Health-Biochemistry and Molecular Biology, Campus Universitario de Puerto Real, University of Cadiz, Puerto Real, Cadiz, 11510 Spain; 2 Biomedical Research and Innovation Institute of Cadiz (INiBICA), Cadiz, Spain; 3 Institute of Biomolecules (INBIO), University of Cadiz, Cadiz, Spain; 4 Institute of Viticulture and Agri-Food Research (IVAGRO) - International Campus of Excellence (ceiA3), University of Cadiz, Cadiz, Spain

**Keywords:** ZNF330/NOA36, AP-MS, HSPA1, HSPA8, ERH, Cell cycle and proliferation

## Abstract

**Background:**

The human genome contains nearly 20.000 protein-coding genes, but there are still more than 6,000 proteins poorly characterized. Among them, ZNF330/NOA36 stand out because it is a highly evolutionarily conserved nucleolar zinc-finger protein found in the genome of ancient animal phyla like sponges or cnidarians, up to humans. Firstly described as a human autoantigen, NOA36 is expressed in all tissues and human cell lines, and it has been related to apoptosis in human cells as well as in muscle morphogenesis and hematopoiesis in Drosophila. Nevertheless, further research is required to better understand the roles of this highly conserved protein.

**Results:**

Here, we have investigated possible interactors of human ZNF330/NOA36 through affinity-purification mass spectrometry (AP-MS). Among them, NOA36 interaction with HSPA1 and HSPA8 heat shock proteins was disclosed and further validated by co-immunoprecipitation. Also, “Enhancer of Rudimentary Homolog” (ERH), a protein involved in cell cycle regulation, was detected in the AP-MS approach. Furthermore, we developed a NOA36 knockout cell line using CRISPR/Cas9n in HEK293, and we found that the cell cycle profile was modified, and proliferation decreased after heat shock in the knocked-out cells. These differences were not due to a different expression of the HSPs genes detected in the AP-MS after inducing stress.

**Conclusions:**

Our results indicate that NOA36 is necessary for proliferation recovery in response to thermal stress to achieve a regular cell cycle profile, likely by interaction with HSPA1 and HSPA8. Further studies would be required to disclose the relevance of NOA36-EHR interaction in this context.

**Supplementary Information:**

The online version contains supplementary material available at 10.1186/s13062-023-00384-8.

## Background

Currently, there are still more than 6,000 human proteins whose function remains unknown [1] (around a 20% of the protein-coding genes), which represents an often-unstudied blind spot that limits the progress of basic and applied biosciences. Indeed, protein characterization has traditionally focused on core biological functions with an immediate impact over those that reflect interactions with the environment, that are usually associated with the age-related accumulation of damaged or misfolded proteins. For instance, most of the protein functions elucidated in the fission yeast between the years 2016 and 2018 were related to environment-responsive processes such as proteostasis, detoxification, mitochondrial organization, or lipostasis [2]. In humans, these processes are mainly relevant in neurodegenerative diseases, such as Alzheimer’s and motor neuron diseases [3], and cancer [4], underlining the importance of studying conserved uncharacterized eukaryotic proteins which will likely have essential cellular functions conserved over several hundred million years of evolution.

ZNF330/NOA36 (NOA36 henceforth) is a remarkable example of such proteins. Initially characterized as a nucleolar human autoantigen [5], NOA36 contains three domains: an N-terminal nucleolar localization signal [6], a poly-acidic C-terminal and a central core of several zinc finger domains. This structure is not only highly preserved in vertebrates but also in invertebrates. For instance, NOA36 orthologous genes have been identified in the genome of a Protista (genus *Thecamonas*) and some species from ancient animal phyla such as Placozoa, Porifera and Cnidaria (Additional file 1).

It is worth noting that the cysteine residues and several sequences of at least five amino acids in NOA36 have not changed in organisms in hundreds of million years of evolution. For instance, amino acids from 146 to 178 in the human NOA36 share an 85% identity with the orthologous sequences of a Placozoan, a sponge, or a hydra species, and a stretch of fourteen identical consecutive amino acids (Additional file 1).

**Fig. 1:**
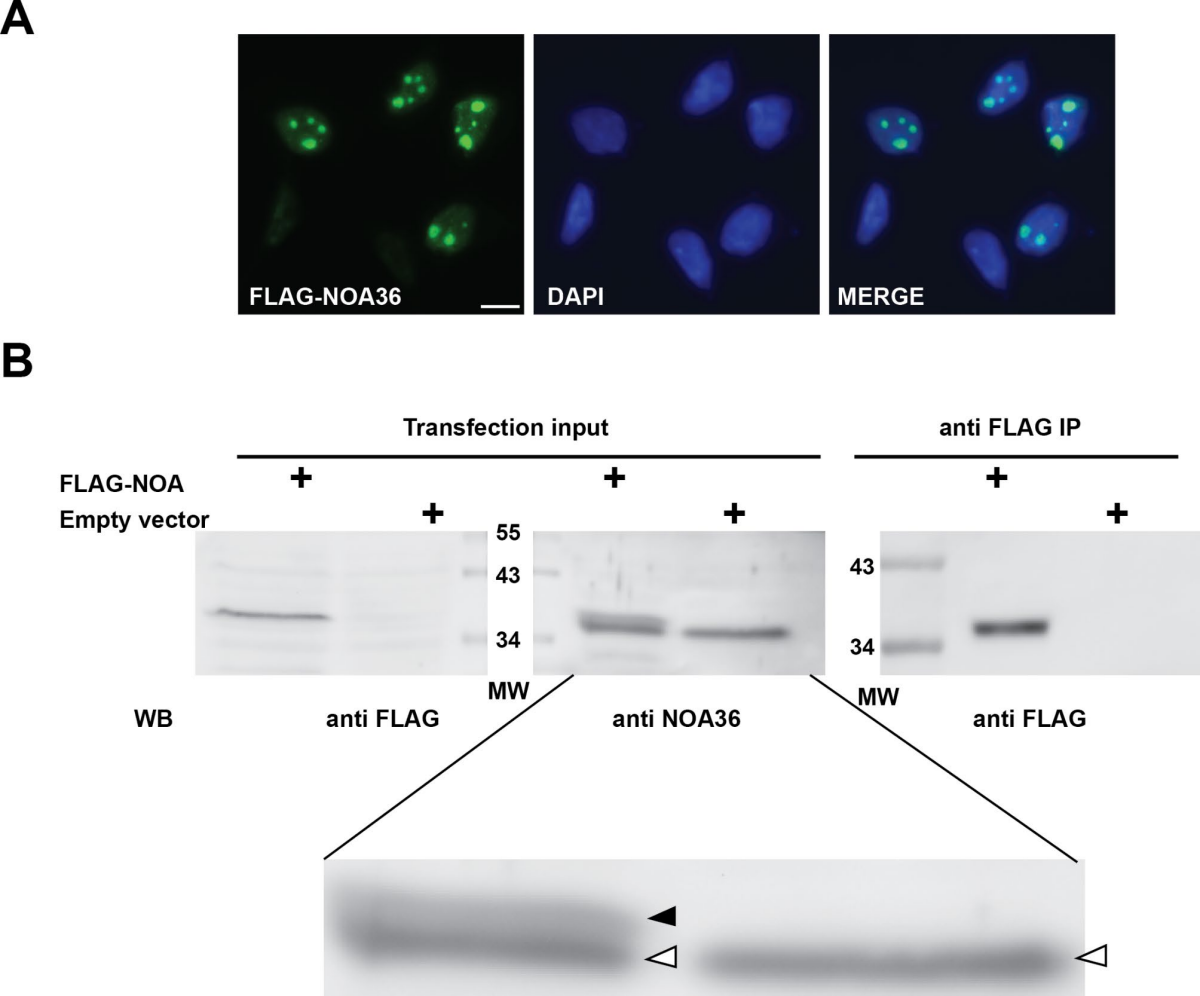
FLAG-NOA36 expression reproduces the endogenous intracellular localization of this evolutionary preserved protein. **(A)** Transfection of HeLa cells with the construct FLAG-NOA36. The expression detected with a specific anti-FLAG antibody by indirect immunofluorescence reproduces the nucleolar localization of the endogenous protein. Bar, 10 μm. **(B)** Western blot analysis of a transfection with the FLAG-NOA36 construct in HeLa cells. FLAG-NOA36 is detected by the anti-FLAG antibody in the transfected cell extract but not in the empty vector transfected one (left blot). A polyclonal anti-NOA36 antibody recognizes both, the endogenous NOA36 (white arrowhead) and the FLAG-NOA36 (black arrowhead) proteins (central blot and magnification bellow). The anti-FLAG magnetic beds successfully pull-down the FLAG-NOA36 recombinant protein (right blot)

In humans, NOA36 expression has been reported in all the tissues and cell lines studied, with high levels in heart muscle and cardiomyocytes [5]. Several intracellular localizations have been described for NOA36, depending on the antibody or the tag used for labelling the recombinant protein. The fusion with GFP, RFP or Cherry fluorescent proteins leads to a cytosolic localization that causes apoptosis in HeLa cells [7]. Among all, the nucleolus is the most consistent subcellular localization reported for NOA36, according to different assays using antibodies [5, 8] or small tags, such as FLAG (Fig. 1A and Additional file 2A) [6] or HA (data not shown).

Besides its well-known role in ribosome biogenesis, the nucleolus also serves as a hub for sensing different cellular stresses and regulating cellular response signals [9–11]. Proteomic analyses have revealed that only a third of the approximately 4,500 proteins interacting with nucleolus are directly involved in ribosome production [12, 13]. In addition, several reports have shown that many ribosome biogenesis factors are also involved in regulating the cell cycle, DNA repair and response to intrinsic and extrinsic stress signals [9, 14]. Thus, proteins like glutamate-rich WD repeat containing 1, which is localized to the nucleolus and is released into the nucleoplasm upon nucleolar stress, suppresses p53 by interacting with ribosomal protein L11 [15]. As another example, the nucleolar GTPase Bms1, a core component of the ribosome small subunit processome, interacts and displaces TTF1 from its binding site called replication-fork-barrier to facilitate the replication fork progression at the S-phase [16].

Regarding NOA36, although little is known about its function, it has been reported as a potential causal gene associated with the survival risk of cervical cancer [17], and high expression of NOA36 is an unfavourable prognostic marker for prostate cancer and favourable for renal cancer [18]. This protein was also identified as a Tyr phosphorylation target in a proteomic study in Jurkat cells [19].

NOA36 may also have a significant role in development since RNAi for the *Drosophila melanogaster* homologue has been identified in two systematic genetic analyses using RNAi libraries in muscle morphogenesis [20] and larval lymph gland hematopoiesis [21]. However, to the best of our knowledge, there are no KO mice models describing the NOA36 role in vertebrate development. Furthermore, NOA36 has been also linked with apoptosis in HeLa cells [7], although the mechanisms of action of this protein are still poorly understood. Thus, the identification of potential interactors with NOA36 could help to further understand the roles of this highly conserved protein. The most common strategy to study large-scale interactomes in human cell lines [22, 23] and in vivo in model organisms [24–26] relies on high-throughput proteomics technologies [27] based on affinity-purification mass spectrometry (AP-MS) approach tagging the protein of interest with specific antibodies [28] or, more often, high-quality monoclonal antibodies to epitope tags such as FLAG [29, 30]. Alternatively, the CRISPR-Cas9 system and its adaptation as a eukaryotic genome-editing tool have also facilitated enormously the characterization of protein functions in cell cultures and whole organisms [31, 32]. Especially since the binding specificity of the Cas9 nuclease has been improved to avoid the off-target nuclease activity of the wild-type enzyme. [33–35].

In this work, we have applied AP-MS to FLAG-NOA36 transfected human cells using a specific anti-FLAG antibody. Also, the development of a NOA36 KO cell line helped us to investigate the NOA36 function.

## Results

### Affinity-purification mass spectrometry using FLAG-NOA36 overexpression

To investigate NOA36 interactions with other proteins, we transfected HeLa cells with a FLAG-NOA36 construct whose expression shows a consistent nucleolar location using a specific anti-FLAG monoclonal antibody (Fig. 1A and Additional file 2A). Western-blot of protein extracts with the same antibody identified a single band, also detected by a polyclonal anti-NOA36 antibody (Fig. 1B). This recombinant protein was successfully immunoprecipitated with anti-FLAG magnetic beads (Fig. 1B). AP-MS was carried with this protein extract and another with the empty vector as a control.

**Fig. 2:**
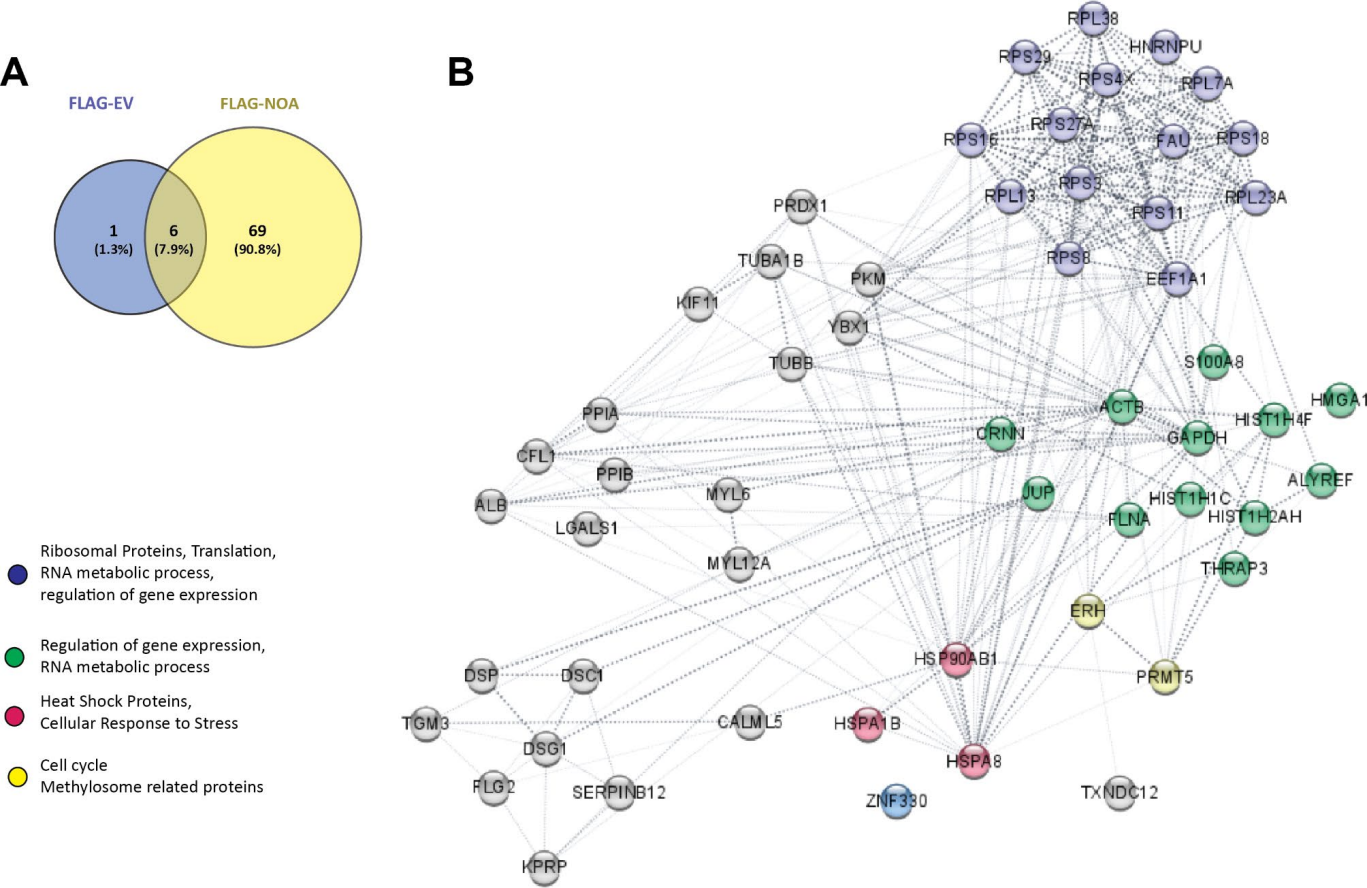
NOA36-interacting proteins detected by AP-MS. **(A)** Venny´s diagram including the number of proteins identified in Hela cells transfected with either FLAG-NOA36 or the FLAG empty vector (as negative control) after the affinity-purification mass spectrometry (AP-MS) assay. The number of common or exclusive proteins identified in both sets of cells is shown. **(B)** A protein interacting network obtained with String platform for the 57 proteins exclusively detected in FLAG-NOA36 transfected cells is shown. Some of the most relevant functions are highlighted

Out of the 75 proteins identified in the NOA36 pull-down sample, six were common to the FLAG empty vector pull-down and therefore discarded for our protein selection (Fig. 2A). In addition, several contaminant keratin proteins were removed from the list, having 57 protein candidates to evaluate (Additional file 3, Table 1).

Among these, we found the Zinc Finger protein 330 (NOA36), confirming the quality of the AP-MS assay. A network was then created with the online STRING Database (string-db.org) to identify potential interaction partners of NOA36 among the identified proteins (Fig. 2B). Several ribosomal-related proteins, including the Elongation factor 1-α-1 (EF1AF1), were detected. These proteins are expected in a nucleolar-enriched extract.

Many nuclear proteins were also found in this panel, including two methyl CpG binding proteins: Enhancer of rudimentary homolog (ERH) and Protein arginine N-methyltransferase 5 (PRMT5) and two histones (H1.2 and H4). ERH is a highly evolutionary preserved protein [36] required for the expression of multiple cell cycle and DNA damage response genes [37]. Analysis of changes in gene expression profile in colorectal cancer cells upon ERH depletion revealed the down-regulation of several additional cell cycle genes [38]. PRMT5 is a highly conserved arginine methyltransferase that, together with CLNS1A, forms the methylosome complex, which modifies specific arginine residues to dimethylarginines in several spliceosomal Sm proteins and histones [39].

Remarkably, three heat shock proteins (HSP) were also identified in the AP-MS analysis: HSPA1, HSPA8, and HSP90AB. We selected these three HSPs to further characterize the interaction with NOA36 because they are also evolutionarily preserved proteins with essential cellular functions.

### Validation of NOA36 interaction with HSPA1 and HSPA8 by co-immunoprecipitation

**Fig. 3:**
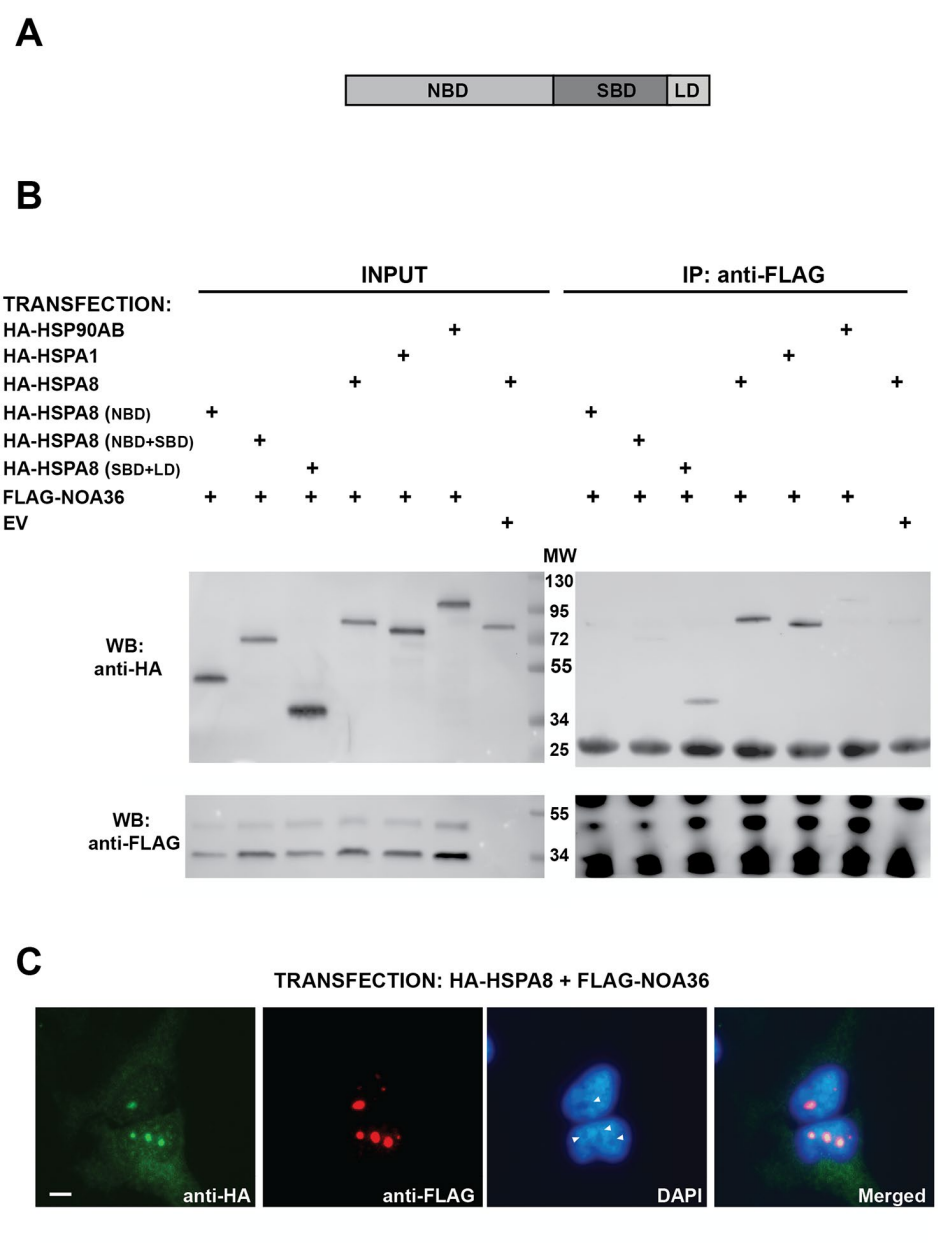
Validation of NOA36 and HSPs interaction by co-immunoprecipitation. **(A)** Scheme of the domains of the HSP70 family of proteins. Three domains have been characterized in these proteins: The Nucleotide Binding Domain (NBD), the Substrate Binding Domain (SBD) and the Lid Domain (LD) **(B)** Western blot analysis of input (left blots) and pull-downs with anti-FLAG magnetic beads (right blots) in HEK293 proteins extracts of cells co-transfected with FLAG-NOA36 and HA tagged heat shock proteins HSP90AB, HSPA1 and HSPA8 (lines 4, 5 and 6). The FLAG empty vector was also co-transfected with HA-HSPA8 as a control (line 7). FLAG-NOA36 co-immunoprecipitates with HA-HSPA1 and HSPA8 but there was not a clear interaction with HSP90AB (upper right blot). Note that the FLAG-NOA36 transfection in HEK293 lead to the expected 36 kDa protein but a 40 kDa band is also detected by the anti-FLAG antibody (bottom left blot). Co-expression of truncated HSPA8 containing the NBD domain (D1, line 1 in the blots), the NBD and SBD (D1 + D2, line 2), and the SBD and LD domains (D2 + D3, line 3). FLAG-NOA36 only clearly interacts with the SBD + LD truncated protein. **(C)** Indirect immunofluorescence of a co-transfection with the constructs FLAG-NOA36 (in red) and HA-HSPA8 (in green) in heat shocked HeLa cells. In blue, DAPI staining showing the nucleoli in the transfected cells (arrow heads). Both recombinant proteins co-localize in the nucleoli of a co-transfected cell (yellow staining in the merged image). Bar, 10 μm

NOA36 consistently localizes in the nucleolus of several human cell lines, including HEK293 cells (Additional file 2B), which were used for characterization of NOA36 due to its high transfection rate. To validate the interaction with NOA36, HA-tagged HSPA1, HSPA8 or HSP90AB recombinant proteins were assayed for co-immunoprecipitation with FLAG-NOA in HEK293 cells with the anti-FLAG antibody. A western blot analysis with an anti-HA antibody showed a strong interaction of NOA36 with HSP1A and HSPA8 but not with HSP90AB (Fig. 3B). The latter was also assayed for immunoprecipitation in HeLa cells, but no interaction between FLAG-NOA36 and HA-HSP90AB was detected (Additional file 4).

Immunofluorescence in HeLa cells showed that the recombinant HA-HSPA8 localized in the cytoplasm of HeLa cells but under heat shock treatment this protein moved to the nucleolus (Additional file 5) and co-localized with FLAG-NOA36 (Fig. 3C and Additional file 6). The endogenous HSPA8 and NOA36 proteins also co-localized in the treated cells (Additional file 7). HSPA8 basic structure (Fig. 3A) includes the three characteristic domains of the HSP70 family: an amino-terminal ATPase binding domain (NBD, amino acids 1-384), a peptide (substrate) binding domain (SBD, residues 385–543), and variable “lid” domain (LD, amino acids 544–646) [40]. The carboxyl-terminal amino acid sequence Glu-Glu-Val-Asp (EEVD motif), which is absolutely conserved in all eukaryotic HSP70 family members, is essential for association with some co-chaperones [41].

To further investigate NOA36-HSPA8 interaction, we constructed three truncated HA-tagged HSPA8 proteins containing the NBD, the NBD + SBD, or the SBD + LD domains. Extracts of HEK293 cells co-transfected with FLAG-NOA36 and each of these three truncated proteins were co-immunoprecipitated with the anti-FLAG antibody. As shown in the Fig. 3B, only the HA-SBD + LD protein strongly binds to FLAG-NOA36. Remarkably, the truncated protein containing both the NBD and SBD did not bind to NOA36.

### Knocking out NOA36 gene in HEK293 cells

To investigate the function of NOA36 in human cells, we knocked out the NOA36 gene in the human HEK293 cell line using CRISPR technology with a nickase Cas9 that avoids the off-target effect of the wild-type nuclease [35] and a pair of sgRNA sequences located at the exon-2/intron-2 boundary. This pair of sgRNA with a six-base par spacer sequence shows no off-target match prediction (Additional file 8A).

To identify NOA36 knockout cells, 48 clones were obtained by limiting dilution and screened by western blot using a specific anti-NOA36 polyclonal antibody [5]. In this screening, two cell lines without NOA36 expression were found (Additional file 8B). We characterized one of them (2D12 cell line) by sequencing the target DNA, finding changes in the coding sequence that introduce premature stop codons that lead to truncated NOA36 proteins expressing only the N-terminal nucleolar localization signal (Additional file 8C and D).

### NOA36 knockout in HEK293 cells alters cell cycle and reduces proliferation after heat shock treatment

**Fig. 4:**
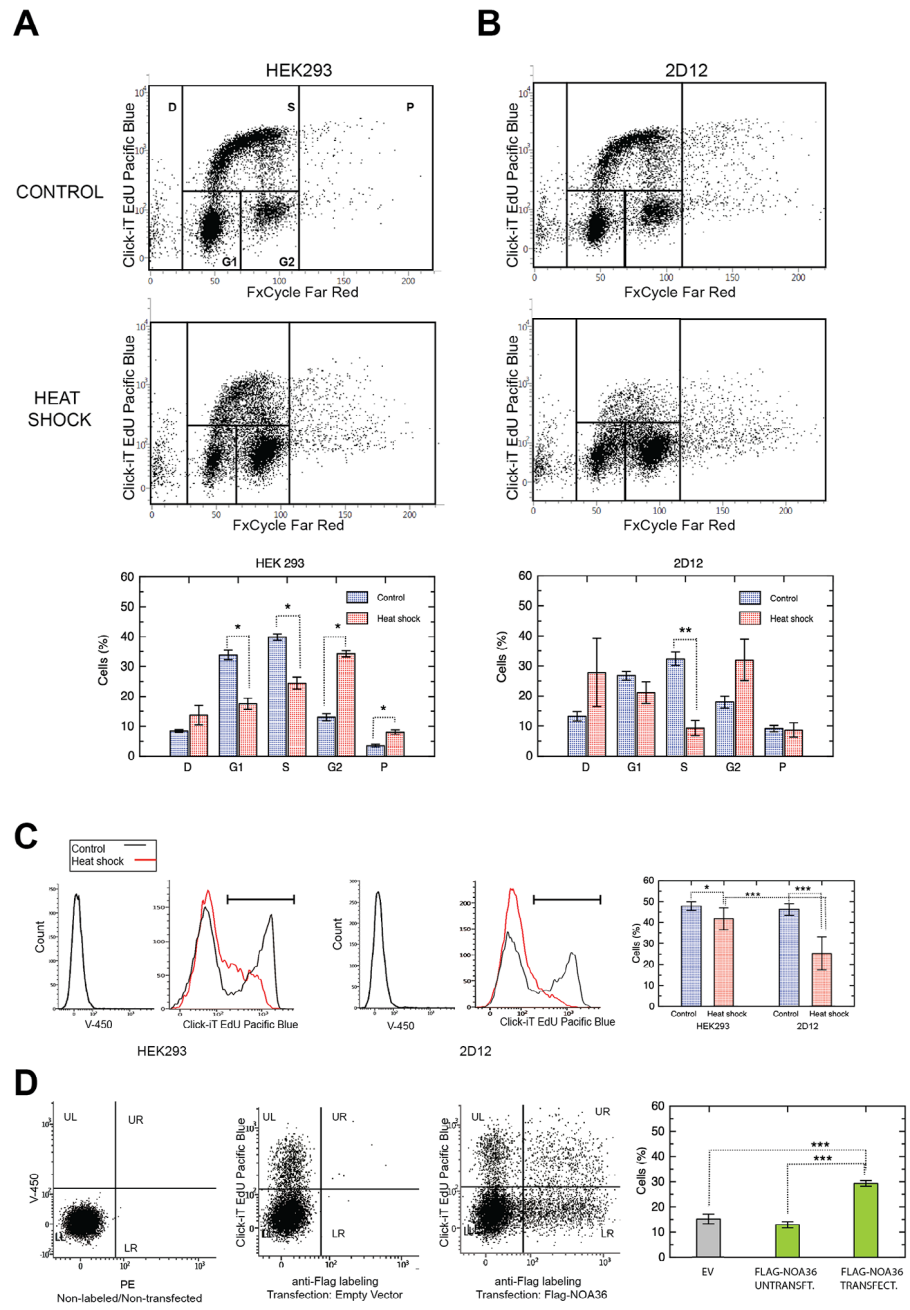
Flow cytometry analysis of cell cycle and proliferation in HEK293 and 2D12 control and heat-shocked cells. **(A)** Cell cycle analysis of HEK293 grown at 37 ºC (control) and 24 h after treatment (heath shock). The percentage of cells in G1, G2, and S phases was measured as indicated in the boxes. The percentage of dead cells -D- and polyploid cells -P- was also calculated. The comparative of these populations (%) is shown in a bar diagram. **(B)** Same analysis in 2D12 cells. **(C)** Analysis of cell proliferation in HEK293 and 2D12 control and treated cells. Flow cytometry histograms of control unlabeled cells (black line) and heat shock treated cells 24 h after treatment (red line). On the right, diagram of the percentage of these populations indicated as the average of seven different replicates. **(D)** Flow cytometry analysis of cell proliferation in 2D12 cells transfected with the FLAG empty vector (central panel) or with the FLAG-NOA36 construct (right panel) 24 h after heat shock. The quadrants represents the following cell populations: LL: non-transfected/non-proliferating cells; UL: non-transfected/proliferating cells; LR: transfected/non-proliferating cells; UR: transfected/proliferating cells. The average percentage in three different experiments for proliferating cells in the control (empty vector, UL + URx100/(UL + LL + UL + UR) in the central panel), the FLAG-NOA36 untransfected cells (ULx100/(UL + LL) in the right panel), and the FLAG-NOA36 transfected cells (URx100/(UR + LR) in the right panel) in three different experiments are shown in the diagram. Left panel, unlabeled cells analysis with the same settings as a negative labeling control. Plots are graphed with the average and standard deviation (SD) with n = 3 in **(A)** and **(B),** and n = 7 in **(C)**. Asterisks denote the statistical significance differences between the groups with three P-values intervals: (*) 0.01 < p < 0.05; (**) 0.001 < p < 0.01 and (***) p < 0.001 using Student’s t-test analysis

Since NOA36 interacts with HSPA1 and HSPA8, which are critical for stress response, and the AP-MS analysis showed that it could also interacts with ERH, a protein involved in the regulation of the expression of several cell cycle proteins, we investigated the effect that the lack of NOA36 expression has on cell cycle after a heat shock stress. To this aim we studied the cell cycle in HEK293 and 2D12 cells by flow cytometry 24 h after heat shock (Fig. 4) and compared them with the corresponding untreated cells as a control.

The cell cycle pattern was altered by the heat shock in HEK293, leading to statistically significant differences between treated and untreated cells, with an increment in the G2 fraction and the reduction of G1 and S phase populations (Fig. 4A). On the other hand, although 2D12 cells showed the same tendency, there were only statistically significant differences in the S phase population between heat shocked and control cells (Fig. 4B). This different behavior was due to a lower proportion of G1 cells in 2D12 control cells (Additional file 9A) and higher variability in G2 population in 2D12 cells than in HEK293 after heat shock (Additional file 9B).

The S cell fraction significantly decreased in both HEK293 and 2D12 after treatment, although in 2D12 it dropped by 71% versus 39% in the case of HEK293. This was the only statistically significant difference after heat shock treatment when both cell lines were compared (Additional file 9B).

In this study we also analyzed the population of dead cells (cell population with lower DNA content than that of the G1 cells) and polyploidy cells (those cells with higher DNA content than those of the G2 population). This analysis showed a tendency toward an increased cell death after heat shock, mainly in 2D12 cells, but there were no significant differences due to the high variance of this population in different experiments. On the other hand, polyploidy was significantly higher in 2D12 control cells (9.2 ± 1.2%) than in HEK293 (3.6 ± 0.5%), while heat shock treatment increased polyploidy in HEK293 but did not change in 2D12 cells (Additional file 9A and B).

Since the more consistent differences were found in the S phase, we analyzed cell proliferation 24 and 48 h after treatment. We found that the proportion of proliferating cells decreased significantly in both cell lines respect to the control untreated cells, although in 2D12 this decrement (45.6%), was higher than that in HEK293 cells (12.3%), (Fig. 4C). Proliferation 48 h after treatment in both cell lines was still lower compared to control cells, although there continued to be significant differences between HEK293 and 2D12 cells subjected to heat shock (Additional File 9C). To study if this effect was due to the lack of NOA36 expression, we also analyzed proliferation in 2D12 cells overexpressing FLAG-NOA36, finding that NOA36 overexpression restored proliferation after heat shock treatment (Fig. 4D).

### NOA36 deletion subtly affects HSPA1 and HSPA8 basal expression but not after heat-shock

**Fig. 5:**
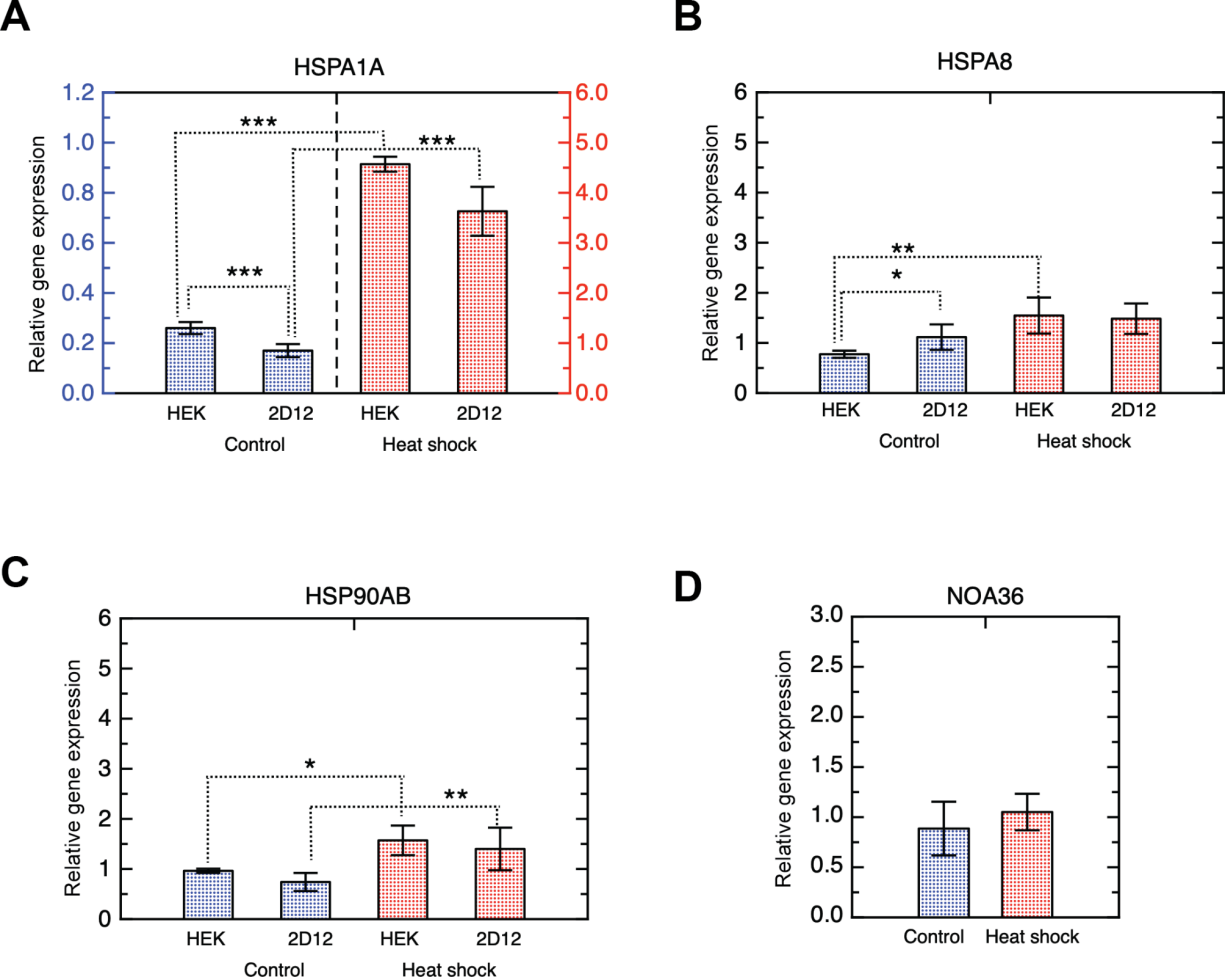
Gene expression analysis of HSPA1, HSPA8, HSP90AB in HEK293. Changes in mRNA expression levels (relative to β-act) for HSPA1A **(A)**, HSPA8 **(B)**, HSP90AB **(C)** and **(D)** NOA36 in HEK293 and 2D12 cells after heat shock treatment (experimental group) and untreated cells (control group). Data are presented as mean ± SD with n = 6. Asterisks indicate different significant different values between the groups with three P-values intervals: (*) 0.01 < p < 0.05; (**) 0.001 < p < 0.01 and (***) p < 0.001

To investigate if the differences in cell cycle and proliferation were due to altered HSPA1, HSPA8 or HSP90AB expression in 2D12 cells, we carried a RT-qPCR analysis of these genes in HEK293 and 2D12 in both, heat shock treated and control cells (Fig. 5). This study was carried out immediately after treatment because differences in gene expression in response to heat shock could fade with time.

We found that there were no significant differences in the expression of these genes between the two cell lines after heat shock, although there were subtle differences in the basal expression of HSPA1 and HSPA8 (control cells).

More specifically, HSPA1 expression strongly increased (around 10 folds) in both HEK293 and 2D12 heat shocked cells (Fig. 5A) showing no significant differences between both cell lines. On the other hand, the basal expression at 37 ºC was significantly lower in 2D12 cells respect to that of HEK293, although the differences in basal expression were very low and did not affect to the induction in response to heat shock. On the other hand, HSPA8 expression increased significantly after treatment in HEK293 but not in 2D12 (Fig. 5B). Again, there were no differences between the two cell lines after heat shock. Finally, HSP90AB expression did not show significant differences between the two cell lines, neither in the treated or control cells (Fig. 5C). We also analyzed NOA36 expression in HEK293, finding no significant difference between control and heat-shocked cells (Fig. 5D).

## Discussion

NOA36 gene has been found in the genome of a unicellular Protista (Additional file 1), which would indicate that it has a function at a cellular level, but NOA36 also has a role in the development of multicellular organisms, as it was demonstrated in some studies in *Drosophila melanogaster* [20, 21]. We previously characterized a nucleolar localization signal in the human NOA36 protein and constructed a vector capable of reproducing the nucleolar localization of the human protein by tagging the FLAG peptide to the N-terminal [6]. Using this construct, we now carried out AP-MS with a highly specific anti-FLAG monoclonal antibody to investigate NOA36 interactors in a non-biased way. Thus, although several proteomics studies have already identified possible human NOA36 interactor-proteins (Q9Y3S2 UNIPROT entry), we disclosed here, for the first time, the interaction of NOA36 with three heat shock proteins (HSPA1, HSPA8 and HSP90AB), and we further validated the interaction of HSPA1 and HSPA8 with NOA36 by co-immunoprecipitation. HSPA1 and HSPA8 interact with each other and can form a stable complex upon heat shock [42]. According with our AP-MS results, NOA36 interacts with this complex. HSPA1 and HSPA8 have a similar structure, with a N-terminal ATPase domain (NBD), substrate-binding domain (SBD), and a C-terminal α-helical “lid” domain (LD). However, HSPA1 and HSPA8 show significant differences in their carboxyl-terminal domain, which is involved in mediating substrate specificity and particular biological functions [43]. NOA36 did not bind to the SBD or a truncated HSPA8 protein lacking the LD, but strongly bound to a truncated protein without the NBD domain. Therefore, HSPA8 would require the LD to retain NOA36 as a substrate or, alternatively, NOA36 would bind to the HSP’s LD, acting as a modulator of HSPA8 activity.

Although there is no experimental data of NOA36 protein structure, the prediction through the AlphaFold software [44] shows that almost the third C-terminal portion of this protein has either a “low” or a “very-low” prediction score, which means that this part of NOA36 may be unstructured in isolation (Additional file 10). Intrinsically, disordered proteins are involved in regulation, signaling, and control, where binding to multiple partners and high-specificity/low-affinity interactions play a crucial role [45].

HSPA8 is constitutively expressed and is required to maintain several cell functions [40], while HSPA1 is mainly responsible for the prevention of protein damage or protein aggregation as well as the reestablishment of functional proteins under stress situations [46]. A proteomic study of nucleoli in HeLa cells revealed that HSPA1 and HSPA8 are nucleolar components in human cells [12]. HSPA8 concentrates in the nucleoli when cells are exposed to stress such as heat shock [47]. In this work, we show that HA-HSPA8 recombinant protein moves to the nucleolus (Additional file 5) and co-localizes with NOA36 (Fig. 3C and Additional file 6). A HSPA8 normal expression [48] and elevated levels of the HSPA1 [49] protect against α−synuclein aggregation, which is linked to the onset and pathology of Parkinson’s disease. Interestingly, both HSPA8 and NOA36 have been reported to be downregulated at least in one study [50].

HSPA1 and HSPA8 have different expression patterns. HSPA8 is only mildly induced during stress, while HSPA1 expression is highly induced during stress [51]. This different expression pattern was corroborated by our RT-qPCR analysis in HEK293 and 2D12 cells in which HSPA1 expression increased around 20 and 15 folds respectively after heat shock induction. It is worth noting that NOA36 interaction with HSPA1 was detected at 37 ºC, a temperature at which this inducible protein shows a very low basal concentration in HEK293 cells (Fig. 5A). High constitutive levels of HSPA1 are well documented in various cancer cells, in which enhances cell growth, suppresses senescence, and confers resistance to stress-induced apoptosis [52]. This analysis also revealed that cells lacking NOA36 show a lower HSPA1 and a higher HSPA8 basal expression, although the differences were very subtle. These results may indicate that, besides a physical interaction with HSPA1 and HSPA8, NOA36 somehow interferes with their transcription. NOA36 structure is compatible with that of a transcription factor, with several zinc finger domains and a poly-acidic C-terminal, a common feature in chromatin-binding proteins. It has been reported that HSPA8 [43] and HSPA1 [53] interact with HSF1 (heat-shock factor 1), a transcription factor that plays an essential role in mediating the appropriate cellular response to diverse forms of physiological stresses. This interaction is a key step in the autoregulation of the heat shock response, and it would be worth investigating a possible interaction of NOA36 with this complex to regulate HSF1.

The AP-MS analysis also disclosed the interaction of NOA36 with ERH, a protein originally identified in *Drosophila melanogaster* that is also highly conserved among metazoans. The ERH gene is required for the expression of multiple cell cycle and DNA damage response genes. Analysis of changes in gene expression profile in colorectal cancer cells upon ERH depletion revealed the down-regulation of several additional cell cycle genes [37, 38].

For this reason, we decided to study the connection of NOA36 with the cell cycle and thermal stress. To this aim, we analyzed G1, S and G2 by flow cytometry in heat-shocked HEK293 cells with and without NOA36 expression. For this, we developed a NOA36 knockout HEK293 cell line by using CRISPR/Cas9n to avoid off-target mutations.

The cell cycle requires proper protein folding after heat shot stress. We found that 24 h after the heat shock treatment, HEK293 cells in G2 increased, avoiding the G2/mitosis-G1 transition as happened in the untreated control cells, in which G1 and S were the largest cell populations. However, in 2D12 cells G1 and G2 populations did not show statistically significant differences from the control untreated cells. The population of S cells lowered in HEK293 and 2D12 cells after heat shock, but the drop of the S fraction was significantly higher in 2D12. Remarkably, the lack of NOA36 expression caused a higher proportion of polyploid cells in untreated cells. All these results indicate that NOA36 has a role in dealing with stress conditions, probably mediated by an interaction with HSPA1 and HSPA8.

## Conclusions

In this work, using AP-MS, we disclose the interaction of NOA36 with the heat shock proteins HSPA1, HSP8A and the cell cycle-related transcription factor ERH. The development of a knockout NOA36 cell line in HEK293 using CRISPR/Cas9n helped us demonstrate that NOA36 is required for normal proliferation and cell cycle profile in response to thermal stress. We also found altered basal transcription levels of HSP8A8 and HSPA1 genes in the knocked-out cells. Further studies would be necessary to disclose the function of this highly evolutionary preserved protein by characterizing the interaction NOA36-EHR and the possible role of NOA36 in the regulation of the expression of HSPs genes.

## Methods

### Cell lines, culture conditions, heat shock treatment and transfections

HeLa, HEK293 and 2D12 (a NOA36-knockout HEK293-cell line generated in this work) cells were cultured in Dulbecco’s modified Eagle’s medium supplemented with 10% fetal bovine serum and 100 U/mL penicillin and 100 µg/mL streptomycin at 37 °C in a CO_2_ incubator. For heat shock treatment, HEK293 and 2D12 cells were incubated at 44 ºC for 2 h. For transfection, cells were seeded onto 60 mm dishes (HeLa) or 6-well plates (HEK293/2D12) (Corning) at a density of 40–50% confluency, 24 h prior to transfection. Cells were transfected using MATra Magnet Assisted Transfection reagent (IBA Lifesciences) at 80–90% confluency following the manufacturer’s recommended protocol.

### Immunostaining

For indirect immunofluorescence staining, cells grown on cover-slips were washed with PBS and fixed in cold acetone for 10 min at − 20 °C. Cells were then washed with PBS and incubated with primary antibodies diluted in PBS 1:100 of immunoaffinity-purified anti-NOA36 IgGs [5]; 1:500 of anti-FLAG M2 (SIGMA); 1:500 of anti-HA rabbit polyclonal antibody (ab9110 from AbCam) or 1:150 anti-HSPA8 rat monoclonal antibody (ab19136 from AbCam) at 37 °C for 45 min, and then washed with PBS for 30 min at room temperature (RT) and incubated with Alexa fluor 488 or 555-labeled secondary antibodies (Molecular Probes) at 37 °C for 45 min. Finally, cover-slips were washed twice in PBS and mounted in PBS-glycerol containing DAPI at 0.1 µg/mL. A Zeiss Axiophot microscope equipped with 10 ×, 20 × and 40 × NA objectives was routinely used. Images were taken using a SPOT Camera (Diagnostic Instruments Inc.) and processed using Adobe Photoshop software.

### Western blot analysis

Protein lysates from total HeLa, HEK293 or 2D12 cells extracts were separated on 4–20% Bio-Rad Mini-PROTEAN® Precast Gels, electrophoretically transferred to nitrocellulose membranes (Bio-Rad Trans-Blot Turbo Transfer System) and subjected to immunoblot analysis with rabbit anti-NOA36 (1:1000), anti-α − tubulin (1:3000 of DM1A from SIGMA) anti-FLAG, (1:1000 of Asp175 from SIGMA), or anti-HA (1:3000 HA; ab9110 rabbit polyclonal antibody from AbCam). Following incubation of the membranes with 1:2000 HRP-coupled secondary antibodies (Cell Signaling), proteins were visualized by WesternBright Quantum (Advansta) in a UVP-biospectrum imaging system.

### AP-MS: Flag-NOA36 immunoprecipitation, sample preparation and LC-MS analysis

Three sets of 1.1 × 10^6^ HeLa cells were seeded in 60 mm plates and transformed 24 h later with either 6 µg of the FLAG-NOA36 construct [7] or the empty FLAG vector as a negative control. For protein extraction, cells were washed with 10 mL of PBS three times 24 h after transfection, and then collected with 500 µL of lysis buffer (0.5% NP40, 150 mM NaCl, 10 mM Tris pH: 7.5, 0.5 mM EDTA, 2 × Protease Inhibitor (Roche), kept at 4 °C for 30 min, and then centrifuged at 20,000 × g for 30 min. at 4 °C. FLAG immunoprecipitation was carried out using 20 µL of anti-FLAG M2 Magnetic Beads from SIGMA, following the manufacturer´s guidelines. Elution was carried out with 100 µL of 0.2 M glycine HCl pH 3.0 and neutralized with 30 µL of TRIS 1 M, pH 8. Samples from cells extracts and the eluted immunoprecipitated extracts (10 µL) were tested by western blot to identify FLAG-NOA36 protein.

The remaining eluted extracts were precipitated in acetone overnight at − 20 °C and recovered by centrifugation at 17,000 × g for 20 min at 4 °C. Protein pellets were resuspended in 8 M Urea in Tris 10 mM (pH 8), reduced with 10 mM dithiothreitol at 50 °C for 30 min and alkylated with 50 mM iodoacetamide for 20 min at RT in the dark. Samples were digested for 4 h at RT with Lys-C enzyme (Promega (V167), USA) (enzyme/ substrate ratio 1:50), and then diluted four times with 50 mM ammonium bicarbonate for further trypsin digestion (Promega, USA) at 37 °C overnight (enzyme/substrate ratio 1:50). The digested peptides were desalted using a SepPak C18 cartridge and dried in a SpeedVac, prior to analysis by mass spectrometry (LC-MS). Peptide samples (approximately 500 ng/sample) were loaded onto a nano-ACQUITY UPLC System (Waters, USA), using a Symmetry 300 C18 UPLC Trap column (180 μm × 20 mm, 5 μm: Waters), together with a BEH130 C18 column (75 μm × 200 mm, 1.7 μm: Waters, USA), which was equilibrated in 3% acetonitrile and 0.1% formic acid. Peptides were eluted directly into an LTQ Orbitrap XL mass spectrometer (Thermo Finnigan, USA) through a nanoelectrospray capillary source (Proxeon Biosystems, Denmark) at 300 nL/min and using a 120 min linear gradient of 3–50% acetonitrile. Spectra were acquired in a data-dependent acquisition mode, with mass resolution of 30,000 at m/z 400. After each survey scan, the six most intense ions above 1000 counts were sequentially subjected to collision-induced dissociation (CID). Precursors with charge states of 2 and 3 were selected specifically for CID and peptides were excluded from further analysis over 60 s using the dynamic exclusion feature. A peak list containing the information of all the features was generated and exported to the Mascot search engine (Matrix Science Ltd., UK). The database search was performed with Mascot v.2.6 against the UniProt database (taxonomy, *Homo sapiens*), with the following criteria: fixed modification: carbamidomethylation (C); dynamic modifications: oxidation (M); precursor mass tolerance 10 ppm; fragment mass tolerance 0.06 Da; enzyme: trypsin and LysC; 2 missed cleavages were allowed. Data filtering was performed using percolator, resulting in 1% false discovery rate. Additional filters were also used: search engine rank 1 peptides and Mascot ion score > 20. Only those proteins with a minimum of two unique peptides were considered as valid identifications. The proteins identified from the cells transfected with either FLAG-NOA36 or the empty FLAG vector groups were compared with Venny 2.1 online software tool (BioinfoGP CNB-CISC) and further analyzed with the String platform (string-db.org) for functional classification.

### Cloning of heat shock proteins (HSPs) cDNAs in a pCMVA-HA vector and western blot validation

Mammalian expression cytomegalovirus promoter-based vector constructs encoding a N-terminal HA tag (HA-pcDNA3, a gift from Dr. Martinez-Barbera) and the HSP90AB, HSPA8, or HSPA1 ORFs, were generated by PCR amplification from human full-length cDNAs purchased from Source BioScience (HSP90AB cDNA clone MGC:10,493, HSPA8 cDNA clone MGC:29,929, and HSPA1A from cDNA clone MGC:1309). The PCR primers used for HSPs cloning are listed in Additional file 2 (Table 2). All these constructs were validated by Sanger sequencing with Applied Biosystem (ABI) genetic analyzer Prism 3730 (StabVida, Lisboa, Portugal). FLAG-NOA36 and HSPs interactions were validated by co-immunoprecipitation of HEK293 cell extracts co-transfected with the FLAG-NOA36 and one of the HA tagged HSP constructs. 1.0 × 10^5^ cells were seeded in 6 wells plates and processed for anti-FLAG IP as described in section above. FLAG-NOA36 and HSPs were identified in the cell and eluted extracts by western blot using mouse anti-FLAG and rabbit anti-HA antibodies.

### NOA36 knock out in HEK293 by CRISPR Cas9n

Two single guide RNAs (sgRNA1 and sgRNA2) targeting sequences at the boundary of NOA36 exon 2 -which codifies for the start codon- and the intron 2 were designed using the Wellcome Sanger Institute Genome Editing (WGE) software [54]. The spacer sequence for this pair of sgRNAs is six base pairs length and no off-target matches were predicted using the “CRISPR PAIR” software from WGE (https://wge.stemcell.sanger.ac.uk//crispr_pair/981137078_981137081). The double-stranded DNA (dsDNA) codifying for these sgRNAs was obtained by annealing two synthetic ssDNAs (StabVida, Lisboa, Portugal) (Additional file 2: Table 2). Each of these synthetic dsDNA was cloned into pX335-U6-Chimeric_BB-CBh-hSpCas9n (D10A) vector, which expresses a human codon-optimized SpCas9 nickase and a chimeric sgRNA expression. This vector was a gift from Feng Zhang (Addgene plasmid # 42,335). The cloning was carried out as described by Ran et al. [55]. Both constructs were transfected in HEK293 cells and 4 days after transfection, cells were dissociated with trypsin and counted on a TC20 Automated Cell Counter (BioRad). A fraction of the cells was seeded by limiting dilution in ten 96 well plates (3 cells/plate). Individual clones were expanded in 1 mL wells and 48 individual clones were tested by western blot with anti-NOA36 rabbit polyclonal antibody to identify those with no NOA36 expression, and anti-tubulin mouse monoclonal antibody (clone DM 1 A, SIGMA) as loading marker. For characterization of the NOA36 mutations, a fragment of 750 pb around the Cas9n target sequences was amplified by PCR using specific primers (Additional file 3, Table 2), and cloned into the pSpark® TA vector (Canvax Biotech). Ten individual clones were selected for sequencing of DNA inserts.

### Cell proliferation and cell cycle analysis by flow cytometry

Flow cytometry analysis was performed in a BD FACSVerse (Beckton-Dickinson) flow cytometer. Cell proliferation and S phase were analyzed with a Click-iT® EdU Pacific Blue™ (Thermo Fisher). 300,000 HEK293 or 2D12 cells were seeded on 6 wells plate wells and cultured for 24 h and then heat shocked (44 °C for 2 h), or kept at 37 ºC as a control. 24 h later EdU reagent (1 µl·mL^− 1^) was added to the culture medium for 2 h and after then trypsinized and collected at 400 × g and washed with BSA 1% in PBS. After centrifugation the cells were fixed and labelled following the manufacturer’s instructions. For cell cycle analysis FxCycle Far Red (Thermo Fisher) (1 µL·mL^− 1^) and 5 µL RNase (20 mg·mL^− 1^) was applied to the labelled cells and incubated in the dark in a rotary shaker for 30 min. For transfection with Flag empty vector or Flag-NOA36 construct, cells were incubated with 1:500 of anti-FLAG M2 (SIGMA) for 30 min at room temperature (RT), washed and then incubated with anti-mouse Alexa fluor 555-labeled secondary antibody (Molecular Probes).

### Gene expression analysis of HSPA1, HSPA8, HSP90AB, and NOA36 in HEK293 and 2D12 cells

To evaluate gene expression for the HSP genes, the following experimental design was used: four experimental groups were set up: HEK293 wild type at 37 °C (group 1), HEK293 wild type at 44 °C (group 2), 2D12 at 37 °C (group 3), and finally 2D12 at 44 °C (group 4). Each group was composed of six biological replicates, starting with the seeding of 200,000 cells per well in six well plates. After 24 h, groups 2 and 4 were subjected to heat shock treatment (44 °C for 2 h) and groups 1 and 2 were kept at 37 ºC for the same time. Immediately after the treatment, all the samples were withdrawn, washed in PBS, re-suspended in 50 µL of ARN*later*™ stabilization solution (Invitrogen, Waltham, USA) and frozen at -20 °C for further processing.

Total RNA of ARN*later* conserved cells was extracted for each sample with the NucleoSpin® RNA XS kit (Macherey-Nagel) following manufacturer’s protocol and original components, this protocol including the on-column DNA digestion using the RNase free DNase provided with the kit. RNA quantity was measured spectrophotometrically at 260 nm wavelength with a Nanodrop ONE (Thermo Scientific). All samples analyzed showed correct ratios of spectral absorbance of A_260/280_ = 2.0-2.2 and A_260/230_ = 1.8-2.0. For cDNA synthesis, 500 ng of total RNA from each sample was used and qScriptTM cDNA Synthesis Kit (Quanta BioSciences) was employed following manufacturer’s protocol. Generated cDNAs were stored at − 20 °C for a period never exceeding one months.

Specific RT-qPCR primers (Additional file 3, Table 3) for HSP genes (HSP90AB, HSPA8 and HSPA1) and NOA36 were designed using the software primer3 (http://frodo.wi.mit.edu/primer3/) based on the cDNA sequences published in GenBank (http://www.ncbi.nlm.nih.gov/nuccore). Specifically, from human HSPA90AB1 (acc. no.: NM_001371238), HSPA8 (acc. no.: NM_153201), HSPA1A (acc. no.: DQ451402) and NOA36 (acc.no.: AJ006591). As reporter gene, human β-actine was used and specific RT-qPCR primers were designed from GenBank database (acc.no.: NM_001101). All RT-qPCR primers were purchased from IDT as desalted.

All qPCR reactions were performed in 10 µL using the qScript cDNA synthesis kit and PerfeCTa™ SYBR®Green FastMix™ (Quantas BioSciences) with 10 ng of cDNA, and 200 nM of each primer. The reaction was carried out in a 96-well plate with the Bio-Rad CFX96 touch Real Time PCR (Bio-Rad) and Manager 3.1 control software (Bio-Rad). Reactions, ran in technical triplicate, were incubated at 95 °C for 5 min, followed by 40 cycles of 95 °C for 15 s and 60 °C for 1 min. A single-peak melting curve was used to check for the absence of primer-dimer artifacts and non-specific amplifications.

To optimize the qPCR conditions, a pull with 20 µL of each cDNA was employed. This pull, named as “calibrator”, was applied in triplicate (10 ng·µL^− 1^, 1 ng·µL^− 1^, 0.1 ng·µL^− 1^ and 0.01 ng·µL^− 1^ of input cDNA) to check the assay linearity and the amplification efficiency. Finally, the assay was linear between 10 ng·µL^− 1^ and 0.01 ng·µL^− 1^ of cDNA per reaction (amplification efficiencies and regression coefficients are shown in Additional file 1: Table S3).

β-actine was used as the internal reference gene for normalizing mRNA expression data, owing its low CT variability as we found during the qPCR runs (not exceeding 0.5 CT differences among different samples). Sample “calibrator” was used as inter-plates standardization. Finally, relative gene quantification was performed using the 2^−ΔΔCt^ method [56].

### Statistical and software analysis

The distribution of continuous data from cell cycle, proliferation and gene expression analysis, were evaluated by the Shapiro-Wilk’s normality test. The statistical significance differences of mean between the groups in cell cycle and proliferation were evaluated by a Student’s t test and Levene’s test was applied to check the differences of variance. The analysis of variance (two-way ANOVA) was carried out for HSPs expression data using HSD Tukey’s post hoc test. All of the statistical analysis were considered significantly different with a P-value of 0.05 using the IBM® SPSS® Statistics Software v.24. All the results were graphed with pro Fit (Quantum Soft) v.7.0 software.

## Electronic supplementary material

Below is the link to the electronic supplementary material.

Supplementary Material 1

Supplementary Material 2

Supplementary Material 3

Supplementary Material 4

Supplementary Material 5

Supplementary Material 6

Supplementary Material 7

Supplementary Material 8

Supplementary Material 9

Supplementary Material 10

## Data Availability

The datasets used and/or analyzed during the current study are available from the corresponding author on reasonable request.

## Abbreviations

NOA36: Nucleolar Autoantigen 36 kDa
AP-MS: Affinity-Purification Mass Spectrometry.
ERH: Enhancer of Rudimentary Homolog
HSP: Heat Shock Protein
NBD: HSP nucleotide binding domain
SBD: HSP substrate binding domain
LD: HSP lid domain
IP: Immunoprecipitation
