# Supplementary material for: ZNF330/NOA36 interacts with HSPA1 and HSPA8 and modulates cell cycle and proliferation in response to heat shock in HEK293 cells
Source: Biol Direct. 2023 May 30;18:26. doi: 10.1186/s13062-023-00384-8 (PMC10228019; doi:10.1186/s13062-023-00384-8)

Additional file 1. Scheme of the NOA36 domains, highlighting evolutionary preservation of the amino in species from different phyla. Vertebrates (*Homo sapiens,* Q9Y3S2); arthropods (*Drosophila melanogaster*, Q9VAU9); mollusks (*Mytilus coruscus*, A0A6J8APA1); annelids (*Dimorphilus gyrociliatus*, A0A7I8VKW0); platyhelminthes (*Macrostomum lignano,* A0A267E040); bryozoans (*Bugula neritina,* A0A7J7J1Y7); cnidarians (*Hydra vulgaris*; T2M2A9); sponges (*Amphimedon queenslandica,* A0A1X7V7P6); placozoan (*Trichoplax adhaerens,* B3RS89); protistas (*Thecamonas trahens*, A0A0L0D4D4). Numbers in the scheme indicates amino acid position in the human protein.

A

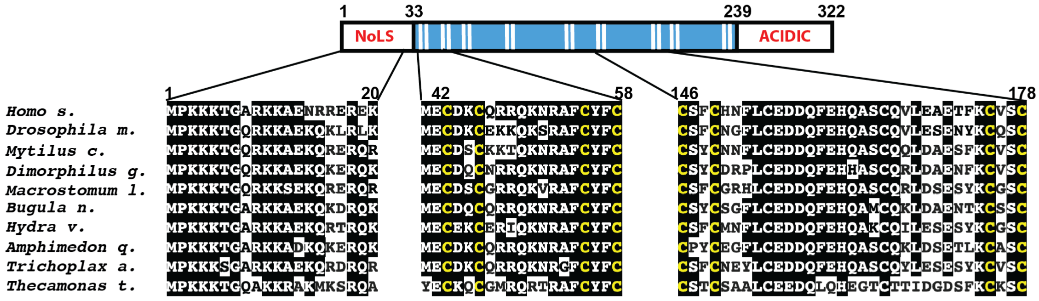

Supplement: Supplementary file 1 — Supplementary Material 1 [file 13062_2023_384_MOESM1_ESM.pdf]
