# Supplementary material for: ZNF330/NOA36 interacts with HSPA1 and HSPA8 and modulates cell cycle and proliferation in response to heat shock in HEK293 cells
Source: Biol Direct. 2023 May 30;18:26. doi: 10.1186/s13062-023-00384-8 (PMC10228019; doi:10.1186/s13062-023-00384-8)

Additional file 2. Indirect immunofluorescence of cells transfected with FLAG-NOA36 in HeLa and HEK293 (A) Pictures taken at low magnification (10 × and 20 × NA) showing multiple HeLa cells expressing nucleolar FLAG-NOA36. (B) HEK293 cells transfected with the construct FLAG-NOA36, showing the nucleolar localization of the recombinant protein (40 x NA).

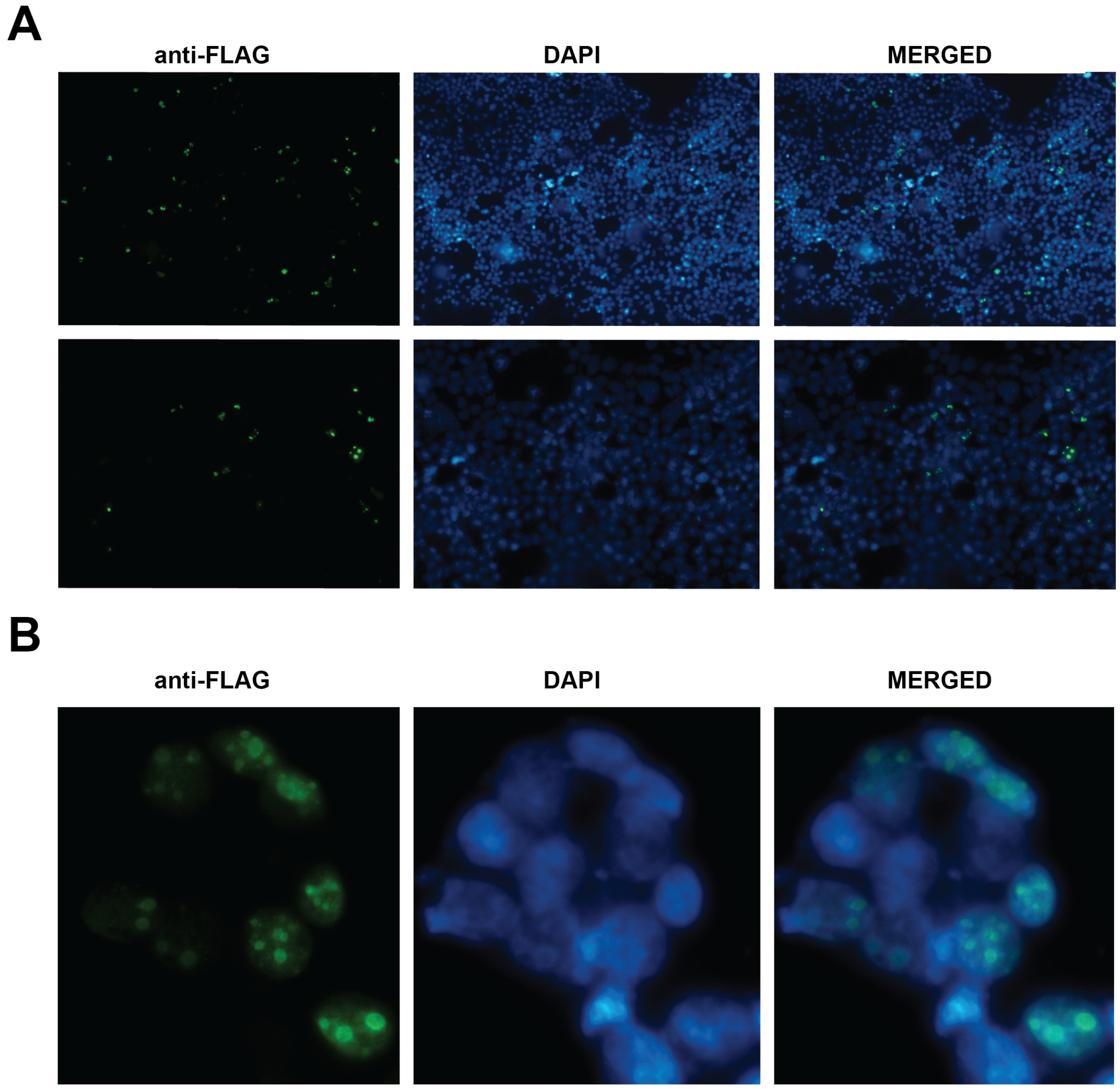

Supplement: Supplementary file 2 — Supplementary Material 2 [file 13062_2023_384_MOESM2_ESM.pdf]
