# Supplementary material for: ZNF330/NOA36 interacts with HSPA1 and HSPA8 and modulates cell cycle and proliferation in response to heat shock in HEK293 cells
Source: Biol Direct. 2023 May 30;18:26. doi: 10.1186/s13062-023-00384-8 (PMC10228019; doi:10.1186/s13062-023-00384-8)
Supplement: Supplementary file 3 — Supplementary Material 3 [file 13062_2023_384_MOESM3_ESM.pdf]

Additional file 3. Table 1. List of the proteins identified by AP-MS after FLAG-NOA36 pull down in HeLa cells. Proteins also identified in the empty vector pull-down, and keratins have been discarded. In bold ZNF/330.

Accession	Gene Name	Description	Score	Coverage %	# Unique Peptides	# Peptides	MW [kDa]	calc. pI
P60709	ACTB	Actin, cytoplasmic 1	258,85	23,73	5	5	41,71	5,48
P23284	PPIB	Peptidyl-prolyl cis-trans isomerase B	226,35	30,09	6	6	23,73	9,41
Q02413	DSG1	Desmoglein-1	214,88	8,48	6	6	113,68	5,03
P02768	ALB	Serum albumin	210,13	19,7	7	7	69,32	6,28
O14744	PRMT5	Protein arginine N-methyltransferase 5	209,04	9,89	6	6	72,64	6,29
Q5T749	KPRP	Keratinocyte proline-rich protein	187,83	11,4	4	4	64,09	8,27
P15924	DSP	Desmoplakin	182,62	2,4	7	7	331,57	6,81
P11142	HSPA8	Heat shock cognate 71 kDa protein	173,73	17,65	7	8	70,85	5,52
P68363	TUBA1B	Tubulin alpha-1B chain	172,14	17,29	5	5	50,12	5,06
**Q9Y3S2**	**ZNF330**	**Zinc finger protein 330**	**170,10**	**15,63**	**3**	**3**	**36,18**	**6,16**
P84090	ERH	Enhancer of rudimentary homolog	163,51	52,88	5	5	12,25	5,92
Q9NZT1	CALM5	Calmodulin-like protein 5	159,63	25,34	2	2	15,88	4,44
P17096	HMGA1	High mobility group protein HMG-I/HMG-Y	152,80	41,12	2	2	11,67	10,32
Q86V81	ALYREF	THO complex subunit 4	149,18	11,28	2	2	26,87	11,15
P04406	GAPDH	Glyceraldehyde-3-phosphate dehydrogenase	148,60	13,73	3	3	36,03	8,46
P14923	JUP	Junction plakoglobin	143,69	13,42	8	8	81,69	6,14
P16403	HIST1H1C	Histone H1.2	139,44	15,49	3	3	21,35	10,93
Q06830	PRDX1	Peroxiredoxin-1	118,68	19,6	4	4	22,10	8,13
P67809	YBX1	Nuclease-sensitive element-binding protein 1	116,46	11,11	2	2	35,90	9,88
P07437	TUBB	Tubulin beta chain	115,04	17,12	5	5	49,64	4,89
Q00839	HNRNPU	Heterogeneous nuclear ribonucleoprotein U	104,51	3,39	2	2	90,53	6,00
P62269	RPS18	40S ribosomal protein S18	102,25	23,68	4	4	17,71	10,99
P05109	S100A8	Protein S100-A8	100,05	23,66	2	2	10,83	7,03
P62241	RPS8	40S ribosomal protein S8	96,22	13,46	2	2	24,19	10,32
P23528	CFL1	Cofilin-1	95,92	25,3	4	4	18,49	8,09
Q9NXV2	KCTD5	BTB/POZ domain-containing protein KCTD5	94,64	17,95	3	3	26,08	6,24
P62979	RPS27A	Ubiquitin-40S ribosomal protein S27a	91,12	18,59	2	2	17,95	9,64
P0DMV8	HSPA1A	Heat shock 70 kDa protein 1A/1B	90,99	6,86	2	3	70,01	5,66
P08238	HSP90AB1	Heat shock protein HSP 90-beta	90,55	5,66	3	3	83,21	5,03
P62750	RPL23A	60S ribosomal protein L23a	88,48	14,1	2	2	17,68	10,45
P23396	RPS3	40S ribosomal protein S3	85,41	13,99	3	3	26,67	9,66

P52732	KIF11	Kinesin-like protein KIF11	81,74	2,56	2	2	119,09	5,64
P01834	IGKC	Ig kappa chain C region	79,05	48,11	3	3	11,60	5,87
P62273	RPS29	40S ribosomal protein S29	76,86	33,93	2	2	6,67	10,13
P19105	MYL12A	Myosin regulatory light chain 12A	75,54	12,28	2	2	19,78	4,81
P62249	RPS16	40S ribosomal protein S16	72,76	21,92	3	3	16,44	10,21
P14618	PKM	Pyruvate kinase isozymes M1/M2	71,40	6,59	3	3	57,90	7,84
P62861	FAU	40S ribosomal protein S30	71,37	20,34	3	3	6,64	12,15
P21333	FLNA	Filamin-A	71,15	1,32	2	2	280,56	6,06
P62701	RPS4X	40S ribosomal protein S4, X isoform	70,71	7,98	2	2	29,58	10,15
Q08554	DSC1	Desmocollin-1	70,02	3,24	2	2	99,92	5,43
Q9UBG3	CRNN	Cornulin	68,97	8,08	2	2	53,50	6,10
P62805	HIST1H4D	Histone H4	68,09	29,13	3	3	11,36	11,36
P62280	RPS11	40S ribosomal protein S11	66,15	15,82	2	2	18,42	10,30
Q08188	TGM3	Protein-glutamine gamma-glutamyltransferase E	62,08	4,47	2	2	76,58	5,86
P60660	MYL6	Myosin light polypeptide 6	58,29	18,54	2	2	16,92	4,65
Q5D862	FLG2	Filaggrin-2	56,61	1,76	2	2	247,93	8,31
Q9Y2W1	THRAP3	Thyroid hormone receptor-associated protein 3	56,40	3,46	2	2	108,60	10,15
P68104	EEF1A1	Elongation factor 1-alpha 1	53,89	7,58	3	3	50,11	9,01
P62937	PPIA	Peptidyl-prolyl cis-trans isomerase A	52,64	16,36	3	3	18,00	7,81
P62424	RPL7A	60S ribosomal protein L7a	49,04	10,15	2	2	29,98	10,61
P26373	RPL13	60S ribosomal protein L13	48,32	8,53	2	2	24,25	11,65
P09382	LGALS1	Galectin-1	44,08	22,22	2	2	14,71	5,50
Q96P63	SERPINB12	Serpin B12	42,05	5,93	2	2	46,25	5,53
Q96KK5	HIST1H2AH	Histone H2A type 1-H	41,59	21,88	2	2	13,90	10,89
P63173	RPL38	60S ribosomal protein L38	36,61	35,71	2	2	8,21	10,10
O95881	TXNDC12	Thioredoxin domain-containing protein 12	33,28	13,95	2	2	19,19	5,40

Additional file 3. Table 2. Oligonucleotides sequences for HSPs cloning into the HA-pcDNA3 vector; guide sequences cloning into p335 CRISPR/Cas9n vector and PCR analysis.

Primer	Sequence (5' to 3')	Direction, description
90AB1-XbaI	CCCTCTAGAATGCCTGAGGAAGTGCACC	Sense, for PCR to clone HSP90AB ORF into HA-pcDNA3.
90AB1-ApaI	CCCGGGCCCCTAATCGACTTCTTCCATGCG	Antisense, for PCR to clone HSP90AB ORF into HA-pcDNA3.
70.1- EcoRI	CCCGAATTCATGGCCAAAGCCGCGGCGATCG	Sense, for PCR to clone HSPA1 ORF into HA-pcDNA3.
70.1-XhoI	TTTCTCGAGCTAATCCACCTCCTCAATGG	Antisense, for PCR to clone HSPA1A ORF into HA-pcDNA3.
70.8-XhoI	GGGCTCGAGATGTCCAAGGGACCTGCAGTTGG	Sense, for PCR to clone HSPA8 ORF into HA-pcDNA3.
70.8-XbaI	GGGTCTAGATTAATCAACCTCTTCAATGGT	Antisense, for PCR to clone HSPA1A ORF into HA-pcDNA3.
gRNA1s-NOA	CACCGATACCATTGAGGCATTACA	Sense, to generate the guide sequence 1 into the sgRNA scaffold in the p335 vector.
gRNA1as-NOA	AAACTGTAATGCCTCAATGGTATC	Antisense, to generate the guide sequence 1 into the sgRNA scaffold in the p335 vector.
gRNA2s+NOA	CACCGTTTTGATATCAGTTGGTAGT	Sense, to generate the guide sequence 2 into the sgRNA scaffold in the p335 vector.
gRNA2as+NOA	AAACACTACCAACTGATATCAAAAC	Antisense, to generate the guide sequence 2 into the sgRNA scaffold in the p335 vector.
up-NOA-tg	GGTGCAGAATACTTTATCATTGGAG	Sense, upstream, to get a 750 pb amplicon around the NOA36 CRISPR target sequence.
lw-NOA-tg	GATGAAAACCCAATAAGACTAAGGC	Antisense, downstream, to get a 750 pb amplicon around the NOA36 CRISPR target sequence.

Additional file 3. Table 3. Oligonucleotides sequences and efficiency parameters for qPCR analysis of HSP90AB, HSPA1, HSPA8 and NOA36 expression in HEK293 and 2D12 cells.

Gene	Primer	Sequence	Efficiency parameters
HSP90AB	h-HSP-90-qPCR F	CGCATGAAGGAGACACAGAA	R^2= 0.995, E= 97.2%
	h-HSP-90-qPCR R	TCCCATCAAATTCCTTGAGC	
HSPA1	h-HSP-70-1-qPCR F	CGACCTGAACAAGAGCATCA	R^2= 0.997, E= 96.5%
	h-HSP-70-1-qPCR R	AAGATCTGCGTCTGCTTGGT	
HSPA8	h-HSP-70-8-qPCR F	GGAGGTGGCACTTTTGATGT	R^2= 0.994, E= 102.3
	h-HSP-70-8-qPCR R	AGCAGTACGGAGGCGTCTTA	
NOA36	h-NOA36-qPCR F	CCAGCTGCCAGGTTTTAGAG	R^2= 0.997, E= 104.1%
	h-NOA36-qPCR R	GCCCACATTTAGGACAAGGA	
β-act.	h-β-act-qPCR F	GGACTTCGAGCAAGAGATGG	R^2= 0.993, E= 95.7%
	h-β-act-qPCR R	CTGTACGCCAACACAGTGCT	
