# Supplementary material for: ZNF330/NOA36 interacts with HSPA1 and HSPA8 and modulates cell cycle and proliferation in response to heat shock in HEK293 cells
Source: Biol Direct. 2023 May 30;18:26. doi: 10.1186/s13062-023-00384-8 (PMC10228019; doi:10.1186/s13062-023-00384-8)

Additional file 4. Co-immunoprecipitation of HA-HSPA8 or HA-HSP90AB with FLAG-NOA36 or the empty vector as a control in HeLa cells. NOA36 co-immunoprecipitates with HSPA8 (line 3) but no with HSPA90AB (line 7). Molecular weight markers (kDa) in line 1 from top to bottom: 130, 95, 72, and 55 in the top panel and 34 and 42 in the bottom panel.

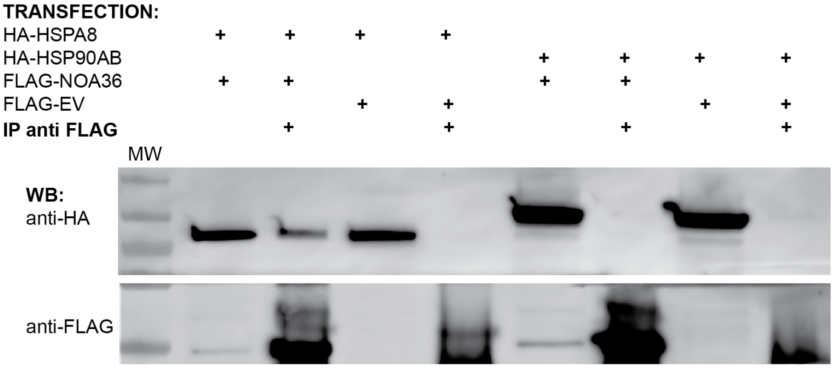

Supplement: Supplementary file 4 — Supplementary Material 4 [file 13062_2023_384_MOESM4_ESM.pdf]
