# Supplementary material for: ZNF330/NOA36 interacts with HSPA1 and HSPA8 and modulates cell cycle and proliferation in response to heat shock in HEK293 cells
Source: Biol Direct. 2023 May 30;18:26. doi: 10.1186/s13062-023-00384-8 (PMC10228019; doi:10.1186/s13062-023-00384-8)

Additional file 5. HA-HSPA8 translocation from cytosol to nucleoli after heat shock treatment in HeLa cells. Top panels: The recombinant protein localizes mainly at the cytoplasm, weakly at the nucleoplasm but no at the nucleoli. Bottom panels: After heat shock treatment a strong nucleolar localization was detected in many transfected cells.

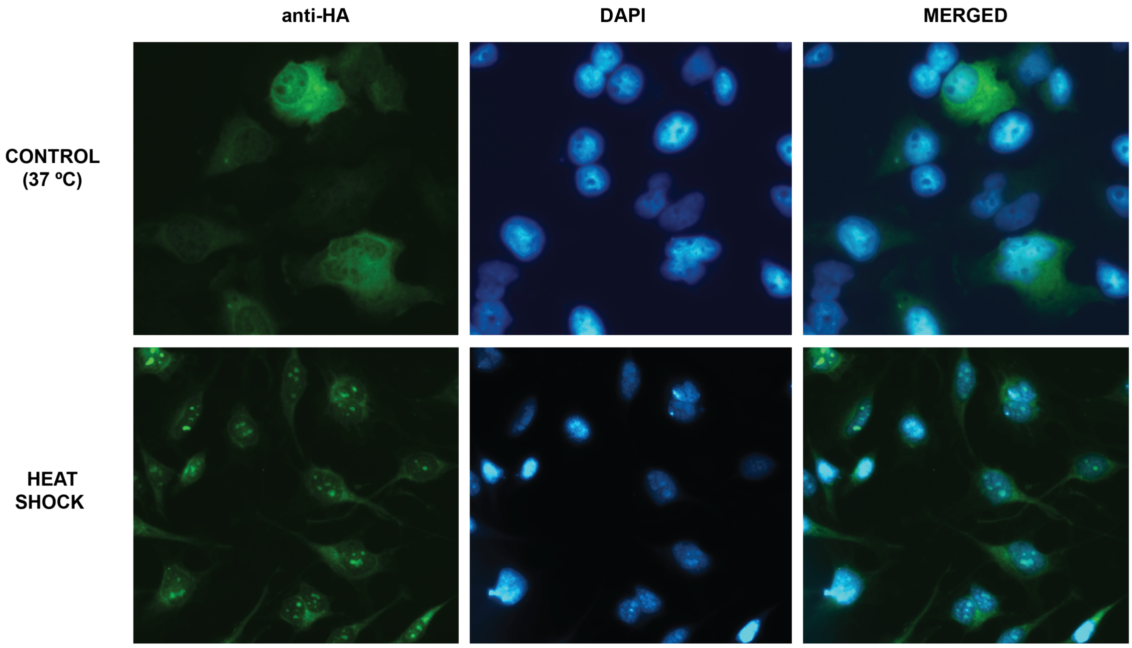

Supplement: Supplementary file 5 — Supplementary Material 5 [file 13062_2023_384_MOESM5_ESM.pdf]
