# Supplementary material for: ZNF330/NOA36 interacts with HSPA1 and HSPA8 and modulates cell cycle and proliferation in response to heat shock in HEK293 cells
Source: Biol Direct. 2023 May 30;18:26. doi: 10.1186/s13062-023-00384-8 (PMC10228019; doi:10.1186/s13062-023-00384-8)

Additional file 6. Several examples of co-localization of FLAG-NOA36 (in red) and HA-HSPA8 (in green) transfected cells in heat shocked HeLa cells. In blue, DAPI staining.

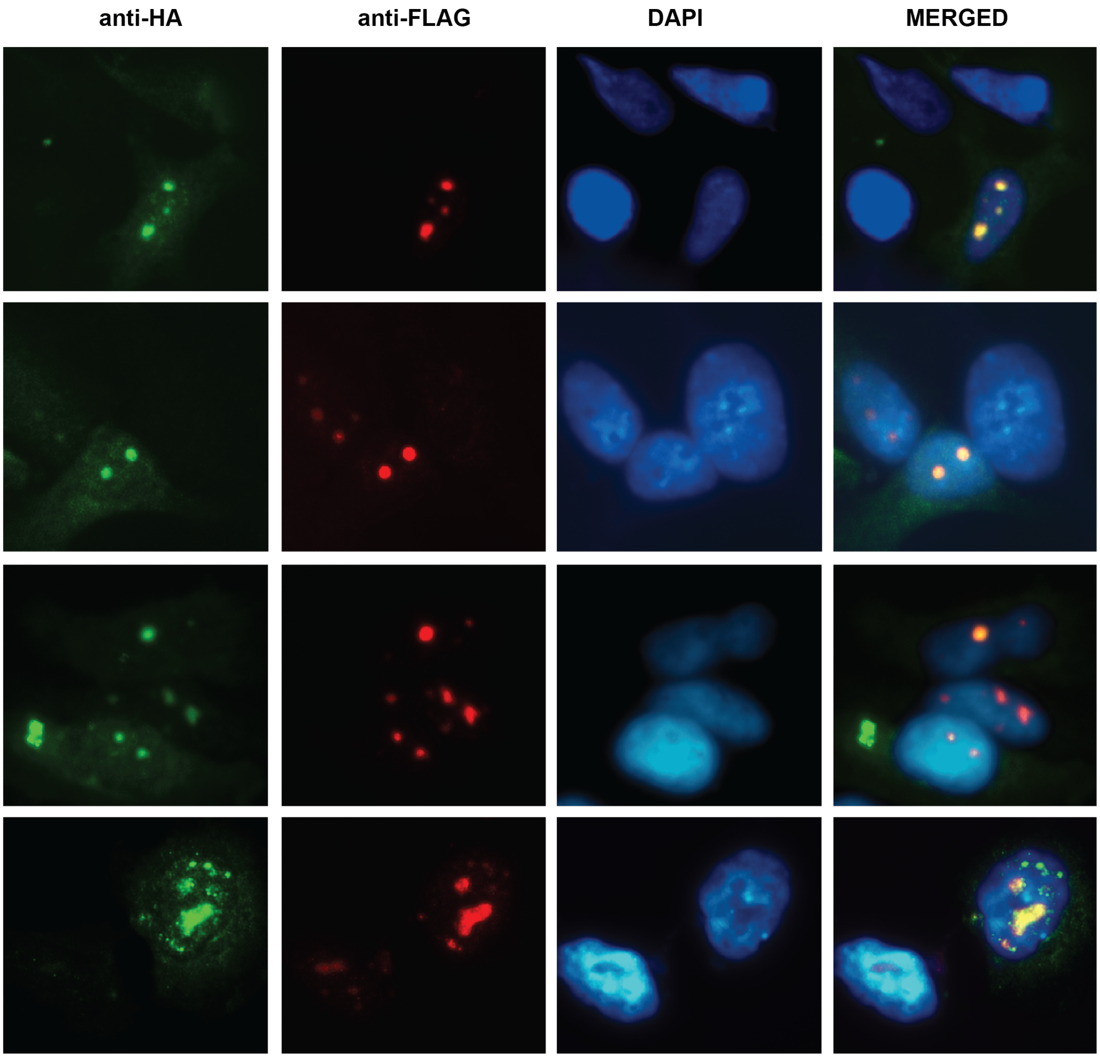

Supplement: Supplementary file 6 — Supplementary Material 6 [file 13062_2023_384_MOESM6_ESM.pdf]
