# Supplementary material for: ZNF330/NOA36 interacts with HSPA1 and HSPA8 and modulates cell cycle and proliferation in response to heat shock in HEK293 cells
Source: Biol Direct. 2023 May 30;18:26. doi: 10.1186/s13062-023-00384-8 (PMC10228019; doi:10.1186/s13062-023-00384-8)

Additional file 7. The endogenous proteins co-localize in the nucleoli after heat shock but not in the control cells. Indirect immunofluorescence with a rat anti-HSPA8 antibody (green) and a rabbit anti-NOA36 antibody in HeLa cells cultivated a 37 ºC and after heath shock.

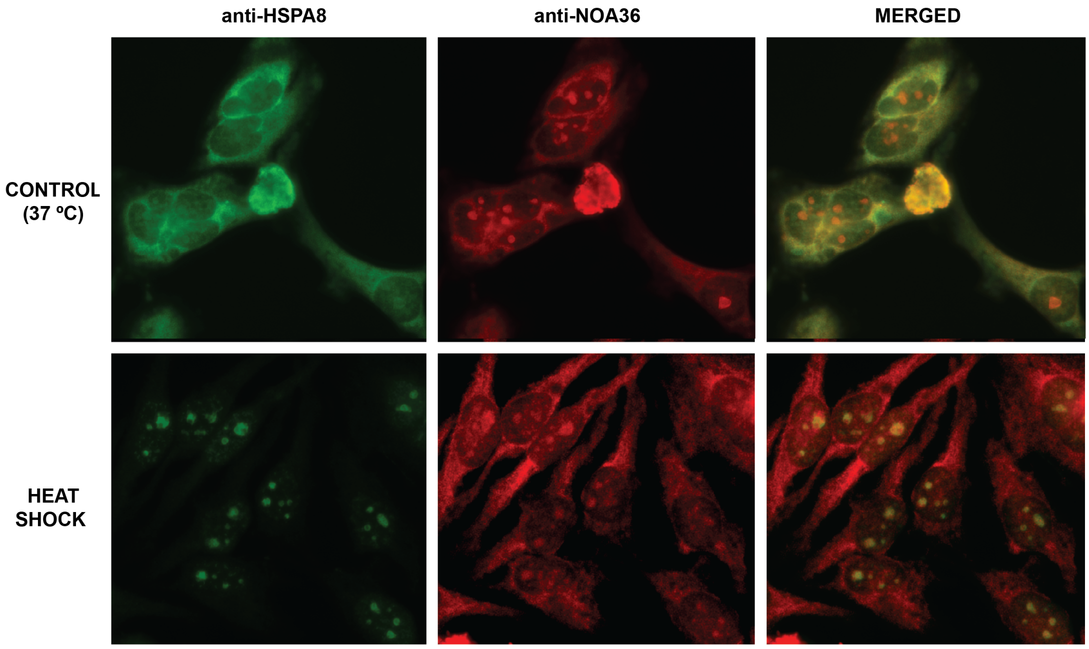

Supplement: Supplementary file 7 — Supplementary Material 7 [file 13062_2023_384_MOESM7_ESM.pdf]
