# Supplementary material for: ZNF330/NOA36 interacts with HSPA1 and HSPA8 and modulates cell cycle and proliferation in response to heat shock in HEK293 cells
Source: Biol Direct. 2023 May 30;18:26. doi: 10.1186/s13062-023-00384-8 (PMC10228019; doi:10.1186/s13062-023-00384-8)

Additional file 6. **Several examples of co-localization of FLAG-NOA36 (in red) and HA-HSPA8 (in green) transfected cells in heat shocked HeLa cells**. In blue, DAPI staining.

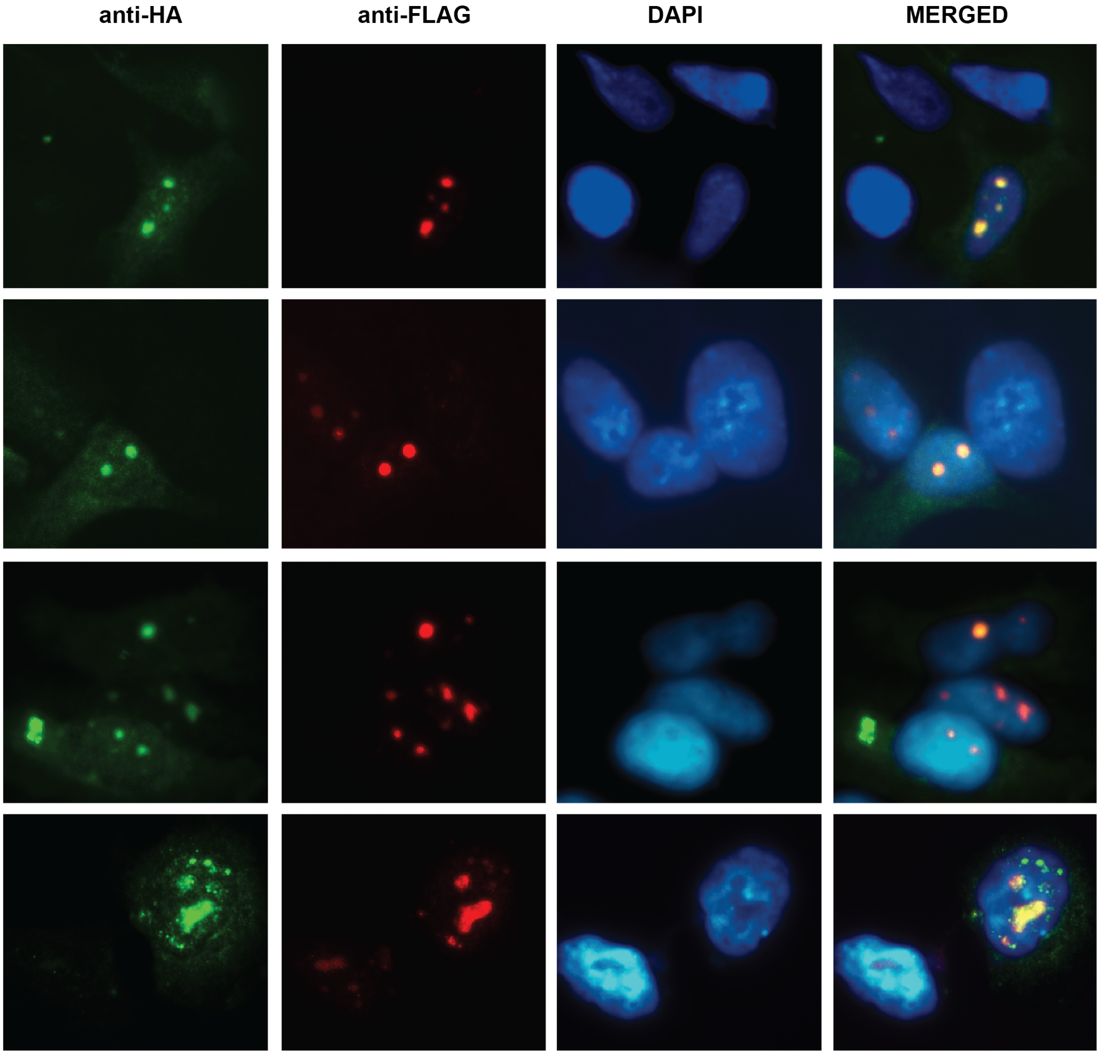

Additional file 7. The endogenous proteins co-localize in the nucleoli after heat shock but not in the control cells. Indirect immunofluorescence with a rat anti-HSPA8 antibody (green) and a rabbit anti-NOA36 antibody in HeLa cells cultivated a 37 ºC and after heath shock.

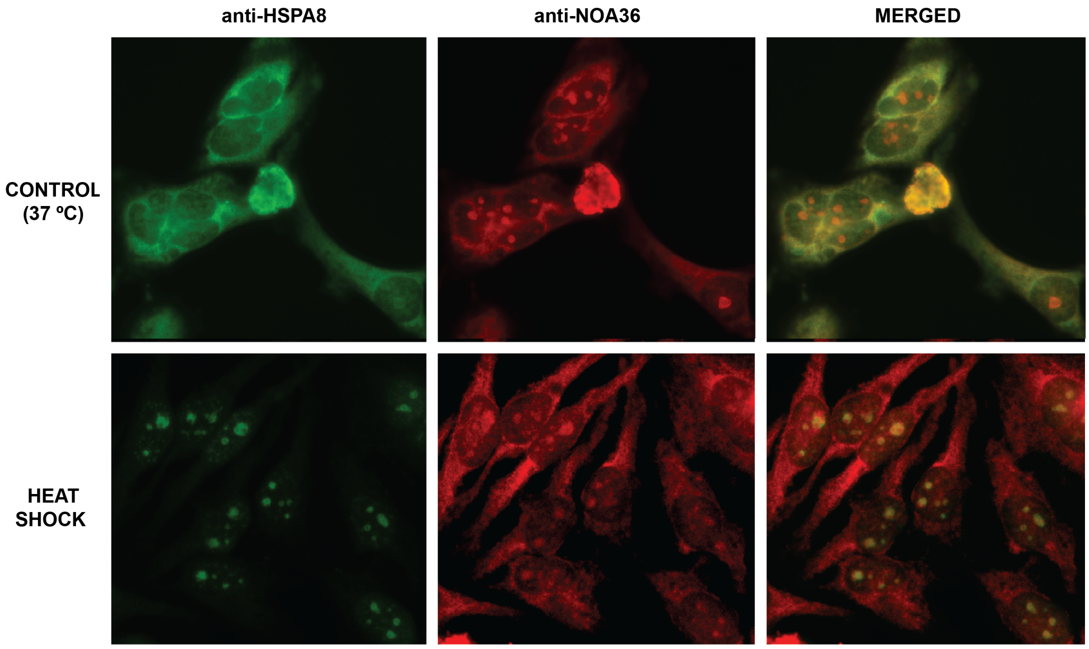

Additional file 8. Development and characterization of a NOA36 knock out HEK293 cell line using CRISPR-Cas9n. (A) Genomic sequence of the second exon (in blue) of the NOA36 gene, which includes the initiation codon (in red). The sgRNA highlighted in light blue were cloned in a vector expressing also a nickase version of the Cas9 nuclease. The predicted cuts are indicated with arrowheads. The PAM sequences are underlined. (B) Western blot test of protein extracts from clones obtained by limiting dilution after transfection with the nCRISPR constructs. A specific rabbit anti-NOA36 polyclonal antibody (36 kDa band) was used to analyze the expression of the target gene. A mouse monoclonal antibody (50 kDa band) was used as a loading control in the same blot. No expression was detected in the 2D12 and 5E3 cell lines. (C) Chromatograms of the wild type HEK293 target sequence and a deletion of 16 base pair (highlighted in yellow in the HEK293 chromatogram) found in the 2D12 cell line. (D) Amino acid sequences of NOA36 N-terminal in the wild type HEK293 and the truncated proteins expressed in the mutated 2D12 cell line. 2D12 expresses the nucleolar localization signal of the NOA36 protein but not the zinc fingers central and polyacidic carboxy terminal domains.

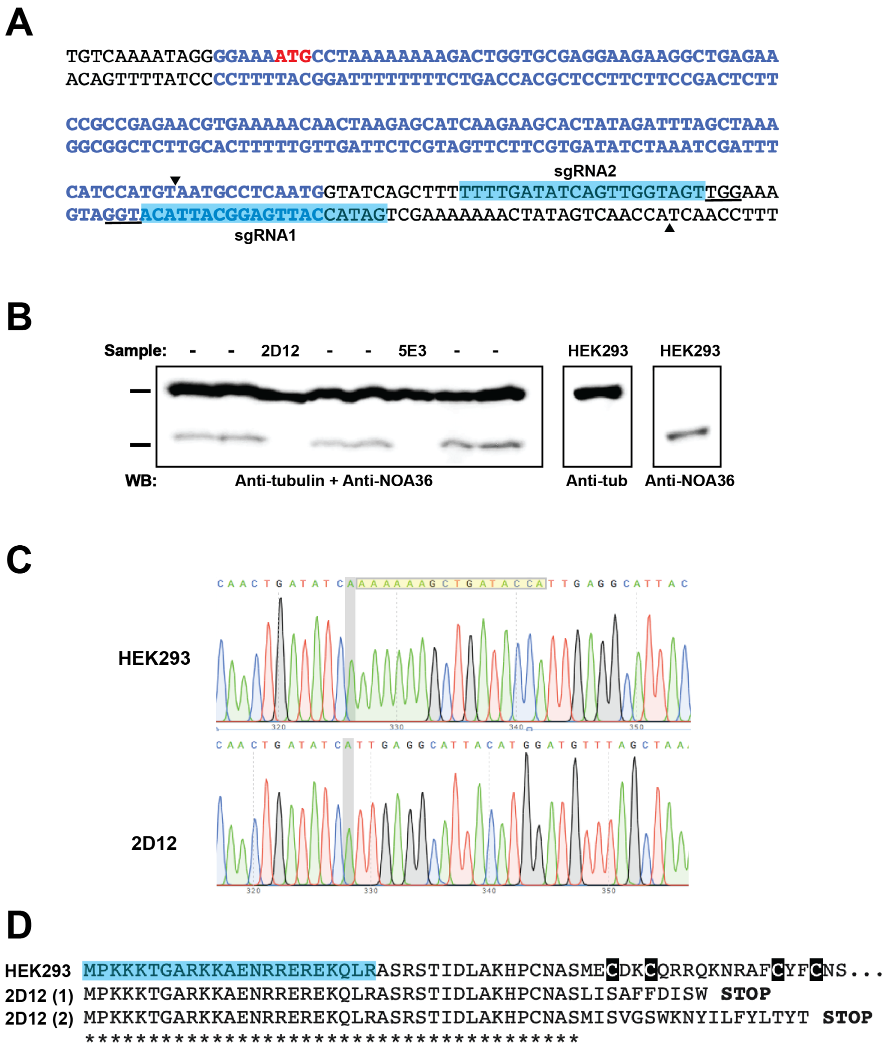

Additional file 9. Comparative analysis of HEK and 2D12 cell cycle profiles from cell flow cytometry results. (A) Control cells grown at 37 ºC. (B) Cells 24 h after heat shock treatment. (C) Analysis of cell proliferation 48 h after heat shock treatment.

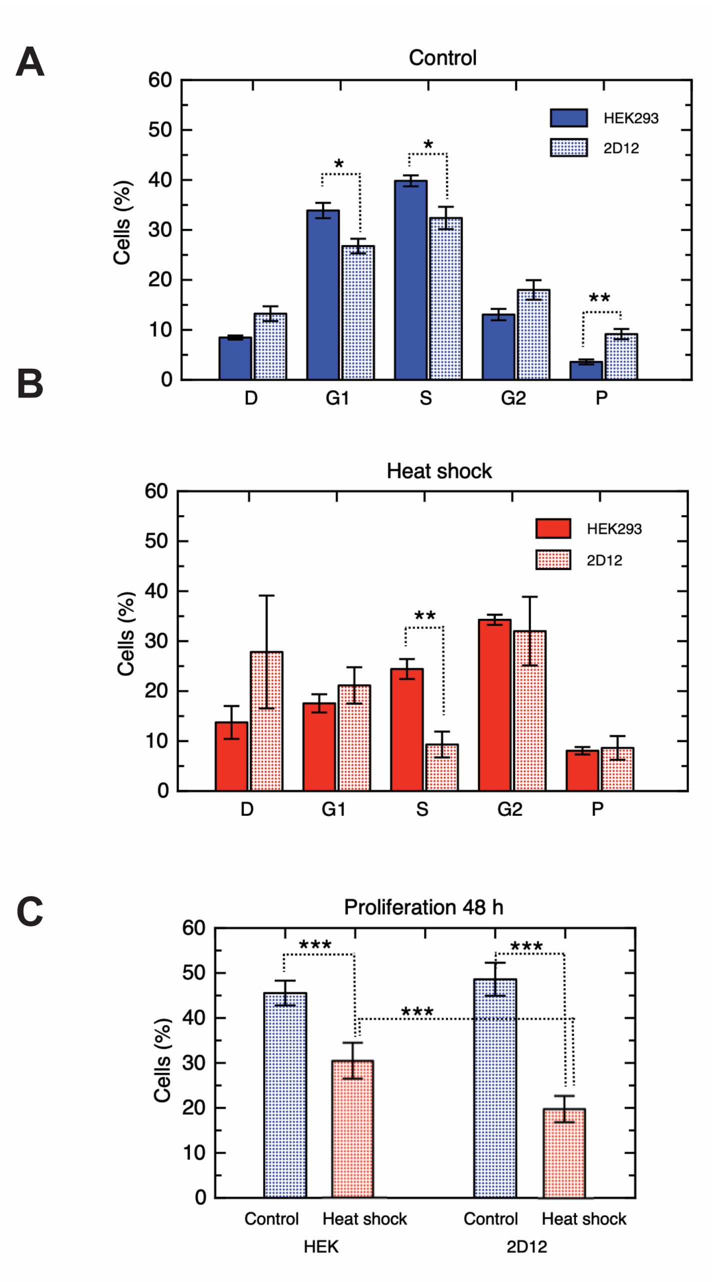

Figure S8. **Structure prediction of the protein NOA36 by AlphaPhold.** The prediction suggests with a high confidence (>70%) that the N-terminal nucleolar localization signal (NoLS) is an α-helix and the central core of zing-fingers (ZFD) is composed of α-helixes and β-sheaths. The C-terminal polyacidic (AD) -around a third of the protein amino acid sequence- has a low o very low confidence prediction, which indicates it may be unstructured in isolation.

Source: UNIPROT (https://www.uniprot.org/uniprotkb/Q9Y3S2/entry).

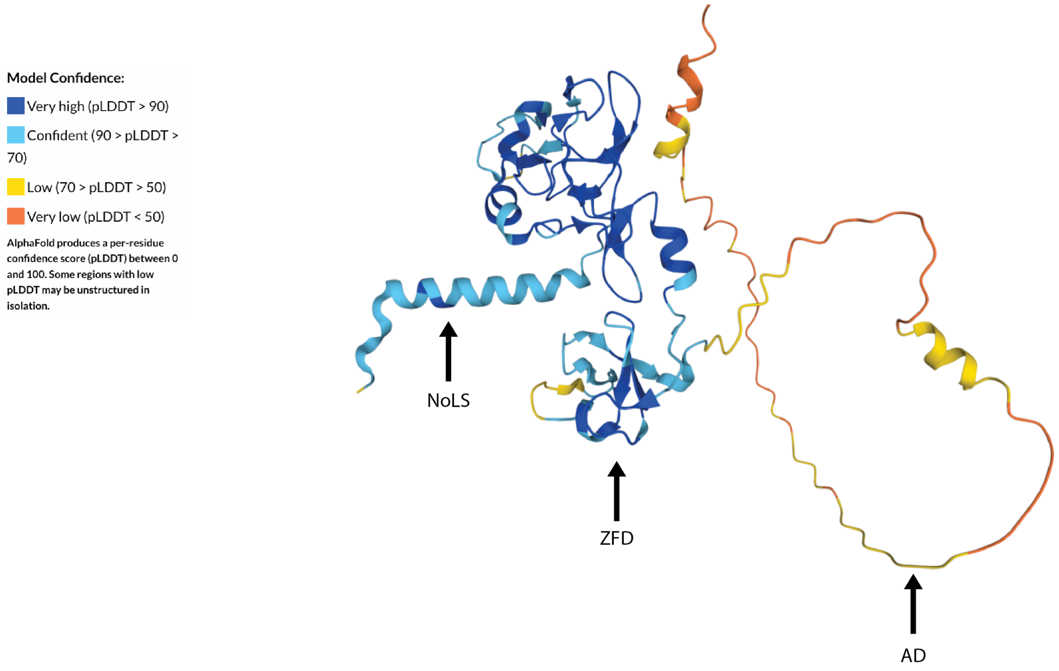

Supplement: Supplementary file 8 — Supplementary Material 8 [file 13062_2023_384_MOESM8_ESM.pdf]
