# Supplementary material for: ZNF330/NOA36 interacts with HSPA1 and HSPA8 and modulates cell cycle and proliferation in response to heat shock in HEK293 cells
Source: Biol Direct. 2023 May 30;18:26. doi: 10.1186/s13062-023-00384-8 (PMC10228019; doi:10.1186/s13062-023-00384-8)

Additional file 9. **Comparative analysis of HEK and 2D12 cell cycle profiles from cell flow cytometry results.** (A) Control cells grown at 37 ºC. (B) Cells 24 h after heat shock treatment. (C) Analysis of cell proliferation 48 h after heat shock treatment.

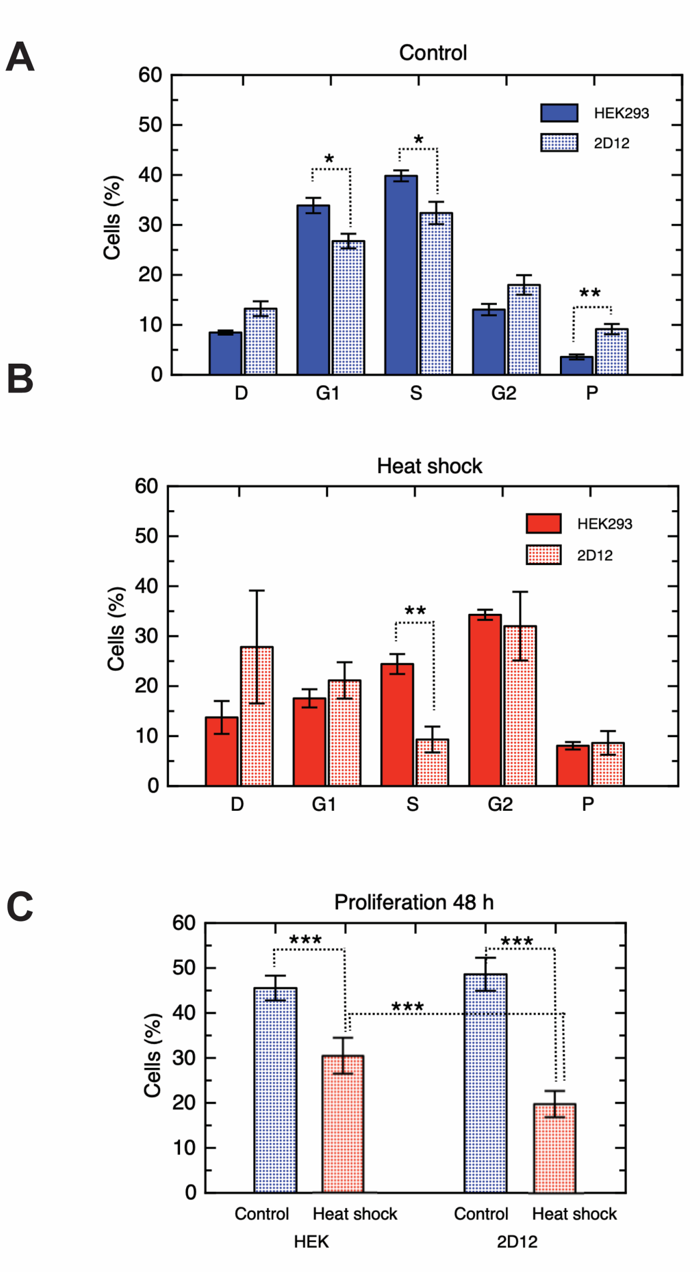

Supplement: Supplementary file 9 — Supplementary Material 9 [file 13062_2023_384_MOESM9_ESM.pdf]
