# Supplementary material for: ZNF330/NOA36 interacts with HSPA1 and HSPA8 and modulates cell cycle and proliferation in response to heat shock in HEK293 cells
Source: Biol Direct. 2023 May 30;18:26. doi: 10.1186/s13062-023-00384-8 (PMC10228019; doi:10.1186/s13062-023-00384-8)

Additional file 10. **Structure prediction of the protein NOA36 by AlphaPhold.** The prediction suggests with a high confidence (>70%) that the N-terminal nucleolar localization signal (NoLS) is an α-helix and the central core of zing-fingers (ZFD) is composed of α-helixes and β-sheaths. The C-terminal polyacidic (AD) -around a third of the protein amino acid sequence- has a low o very low confidence prediction, which indicates it may be unstructured in isolation.

Source: UNIPROT (https://www.uniprot.org/uniprotkb/Q9Y3S2/entry).

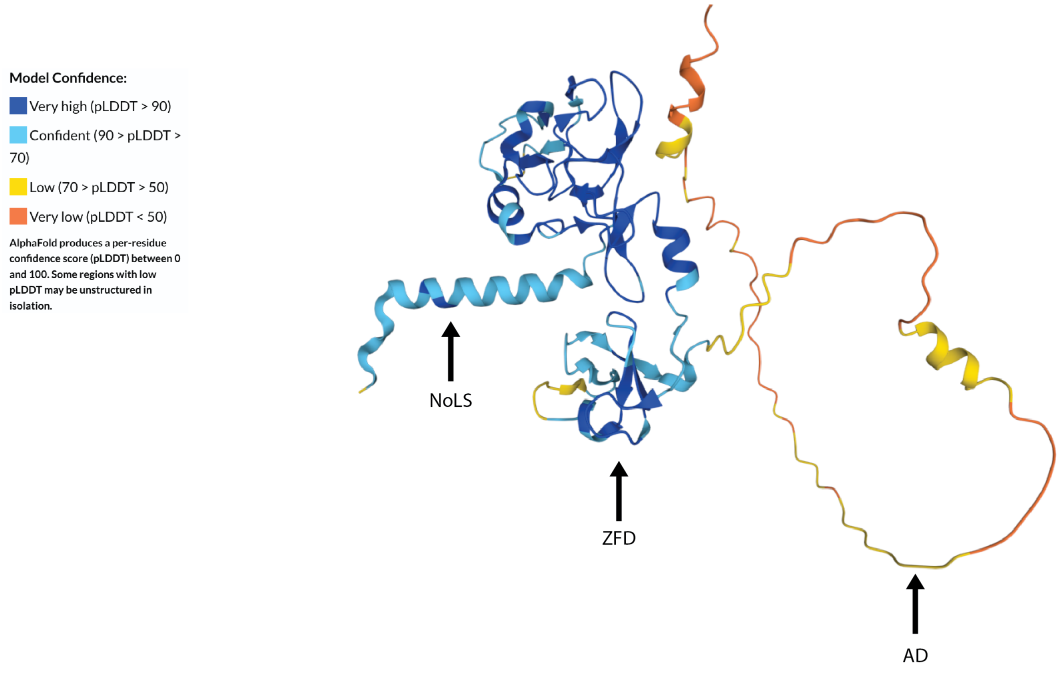

Supplement: Supplementary file 10 — Supplementary Material 10 [file 13062_2023_384_MOESM10_ESM.pdf]
